# Exploring the potential of 3D Zernike descriptors and SVM for protein–protein interface prediction

**DOI:** 10.1186/s12859-018-2043-3

**Published:** 2018-02-06

**Authors:** Sebastian Daberdaku, Carlo Ferrari

**Affiliations:** Department of Information Engineering, University of Padova, via Gradenigo 6/A, Padova, 35131 Italy

**Keywords:** Protein–protein interface prediction, 3D Zernike Descriptors, SVM

## Abstract

**Background:**

The correct determination of protein–protein interaction interfaces is important for understanding disease mechanisms and for rational drug design. To date, several computational methods for the prediction of protein interfaces have been developed, but the interface prediction problem is still not fully understood. Experimental evidence suggests that the location of binding sites is imprinted in the protein structure, but there are major differences among the interfaces of the various protein types: the characterising properties can vary a lot depending on the interaction type and function. The selection of an optimal set of features characterising the protein interface and the development of an effective method to represent and capture the complex protein recognition patterns are of paramount importance for this task.

**Results:**

In this work we investigate the potential of a novel local surface descriptor based on 3D Zernike moments for the interface prediction task. Descriptors invariant to roto-translations are extracted from circular patches of the protein surface enriched with physico-chemical properties from the HQI8 amino acid index set, and are used as samples for a binary classification problem. Support Vector Machines are used as a classifier to distinguish interface local surface patches from non-interface ones. The proposed method was validated on 16 classes of proteins extracted from the Protein–Protein Docking Benchmark 5.0 and compared to other state-of-the-art protein interface predictors (SPPIDER, PrISE and NPS-HomPPI).

**Conclusions:**

The 3D Zernike descriptors are able to capture the similarity among patterns of physico-chemical and biochemical properties mapped on the protein surface arising from the various spatial arrangements of the underlying residues, and their usage can be easily extended to other sets of amino acid properties. The results suggest that the choice of a proper set of features characterising the protein interface is crucial for the interface prediction task, and that optimality strongly depends on the class of proteins whose interface we want to characterise. We postulate that different protein classes should be treated separately and that it is necessary to identify an optimal set of features for each protein class.

**Electronic supplementary material:**

The online version of this article (10.1186/s12859-018-2043-3) contains supplementary material, which is available to authorized users.

## Background

Proteins carry out a broad range of functions in living organisms such as structural support, signal transmission, immune defence, transport, storage, biochemical reaction catalysis and motility processes. The majority of proteins does not act in isolation: in fact they express their biological roles by interacting with other molecules [1]. Protein–protein interactions (PPIs) are of particular interest as they tell us how proteins come together to construct metabolic and signalling pathways in order to fulfil their functions [2]. Dysfunction or malfunction of pathways and alterations in protein interactions have shown to be the cause of several diseases such as neurodegenerative disorders [3] and cancer [4], and hence the identification of the exact location on a protein’s surface where it is likely to bind to its partners, i.e. the binding interface, has become one of the most popular targets for rational drug design [5]. In addition to practical applications, reliable identification of protein–protein interfaces is an important goal for basic research on the mechanisms of macromolecular recognition. For instance, PPI interface predictions can greatly aid protein–protein docking algorithms by being used in scoring functions or to constrain the available search space [6–8].

There are several experimental techniques available which can be employed for the characterisation of protein–protein interfaces at residual and even atomic level. For instance, both X-ray crystallography [9, 10] and nuclear magnetic resonance (NMR) spectroscopy [11] have been used to determine protein interfaces at atomic level. Cryo-electron microscopy [12] has increasingly gained popularity as it allows the examination of native structural features of hydrated molecules in solution. Other techniques provide structural elucidation of interactions at lower resolutions. Alanine scanning mutagenesis [13], Hydrogen/Deuterium exchange [14] and chemical cross-linking [15] have been used to experimentally characterize protein–protein interfaces at residue level.

Although impressive progress has been made, there are several limitations to the existing experimental methods in the determination of protein–protein interfaces. X-ray crystallography requires crystallizing the specimen and placing them in non-physiological environments, which can be inherently difficult and occasionally lead to functionally-irrelevant conformational changes. NMR spectroscopy is suitable for macromolecules in solution (closer to real functional environments or foldings) and can yield information on the dynamics of various parts of a given the protein or complex, but its applicability is limited to small polypeptides (less than 50 kDa). Cryo-electron microscopy has no sample size constraints and can guarantee a reduced radiation damage to the sample compared to X-ray crystallography, but is generally more difficult, time consuming, and requires operating constantly at temperatures lower than –135°C. These technical challenges make such experiments both labour-intensive and time-consuming, while on the other hand, the ongoing proteomics and structural genomics research continues producing large amounts of data, which need to be interpreted in a timely manner. Efficient computational methods are therefore needed to correctly predict the potential binding sites for a deeper understanding of PPIs.

Several computational methods for the prediction of PPI sites are available to date [16] which can be roughly categorised into sequence-based and structure-based approaches [17, 18]. In sequence-based methods, a sliding window of fixed length (typically varying from 3 to 30 residues) is scanned across the protein sequence and a number of overlapping local sequence segments are extracted. For each of these segments, a feature vector is constructed using various amino acid properties (physicochemical, statistical and structural features), and is used as the input of a classification problem. These methods are particularly useful as they allow the PPI site prediction when a protein’s structure information is not yet available.

In [19], a two-stage classifier is employed consisting of a Support Vector Machine (SVM) and a Bayesian network classifier that identifies interface residues primarily on the basis of sequence information. A 9-residue-long sliding window is employed, which is encoded using a 20 bit per residue feature vector (180 bit) for the first stage, and a 1 bit per residue (excluding the central one) feature vector (8 bit) for the second stage. In [20], a sliding window approach is combined with a Random Forests classifier to predict protein interaction sites using sequence information, both alone and in combination with structure-derived parameters. The input feature vectors were derived using a window length of 9 residues and employing 17 features per residue. Murakami and Mizuguchi predict interaction sites in protein sequences with a Naïve Bayes classifier using sequence features only: a position-specific scoring matrix (PSSM) and the predicted accessibility [21]. In [22], 24 independent neural network models are built using sparsely encoded sequence features for each amino acid (20-dimensional binary encoding for each residue) and a PSSM, and the average score of the 24 predictors is returned as the final score. Sriwastava et al. employ 21-residue-long local sequence segment pairs of protein sequences to identify interaction sites in protein complexes [23]. The input samples are built by assigning 8 properties to each residue in the local sequence segment pair, yielding 2×21×8=336-dimensional feature vectors classified by an SVM. In [24], a wide range of features (physicochemical properties, evolutionary conservation, amino acid distances and a PSSM) is extracted from protein sequences without using any structure data, then, a random forest-based integrative model is employed to effectively utilize these features and to deal with imbalanced data. Garcia-Garcia et al. propose a sequence-based computational method that infers possible interacting regions between two proteins by searching minimal common sequence fragments of the interacting protein pairs [25]. A two-dimensional matrix is derived by computing a score for each pair of residues that relates to the presence of similar regions in interolog protein pairs. The potential interface regions are reflected in query proteins by representing the scoring matrix as a heat map.

Structural features associated with the atomic coordinates of proteins are important discriminative attributes for PPI interface prediction, and the absence of such information is therefore expected to reduce the performance of sequence-based predictors compared to structure-based ones. For instance, most interface residues are also located on the protein surface, so structure-based methods can simply identify surface residues and ignore all internal residues. PPI interfaces are comprised of residues that can be located close to each-other in 3D space, while having distant positions in the primary sequence of the proteins. Finally, geometrical complementarity can be evaluated from 3D structures. Structure-based computational approaches offer several advantages over sequence-based ones, but are limited by the availability of protein 3D structures. However, the number and quality of available protein 3D structures has been steadily increasing over the past years and several structural repositories are available to date (i.e. Protein Data Bank (PDB) [26], The PeptideAtlas Project [27], Global Proteome Machine Database (GPMD) [28], The Proteomics Identifications database (PRIDE) [29]), enabling the development of structure-based interface predictors. Currently, most structure-based machine learning interface predictors exhibit better performance than sequence-based methods [16].

Porollo and Meller use “fingerprints” derived from the difference between the predicted and actual relative accessible surface area (rASA) of residues as features for interface prediction [30]. The prediction of PPI sites is done by a consensus method that combines the output of 10 Neural Networks with majority voting. Kufareva et al. developed an alignment-independent method of PPI interface prediction from local statistical properties of the protein surface at the atomic-group level [31]. The classification is done using a partial least-squares regression algorithm on the solvent accessibility values of 12 significantly over-represented and under-represented atomic groups at the interface, and can be further complemented by evolutionary conservation scores. In [32], interface regions for a query protein are determined by clustering and ranking the known interfaces in structural homologs. Zhang et al. propose a structural homology-based PPI interface prediction method [33]. For each query protein, its structural neighbours are identified by structural alignment, and their interface is mapped onto the query protein structure. The frequency of the mapped contacts are calculated for each residue in the query protein, and a logistic function is used to normalize the contact frequencies and generate the final prediction score for each residue. In [34], information from both proteins in a complex is used to predict pairs of interacting residues from the two proteins. Sequence (PSSM and predicted rASA) and structure (rASA, residue depth, half sphere amino acid composition, protrusion index) information about residue pairs is captured through pairwise kernels that are used for training a SVM classifier.

Experimental evidence supports the hypothesis that the location of binding sites is imprinted in the structures of proteins, and that this information can be extracted even without the knowledge of the binding partner [17, 35]. Interface surface portions share common physicochemical properties which distinguish them from the non-interface ones, thus, only specific areas of the protein surface are amenable to be engaged in PPIs. It has been observed that interaction sites are characterised by a high number of *hot spots*, i.e. energetically critical residues that contribute significantly to the free energy of binding [36]. Clusters of hydrophobic residues [37] and aromatic side chains [38, 39] are more abundant in the binding site, while hydrophilic residues are infrequent. Aromatic residues can form strong hydrophobic interactions between the bulky hydrophobic side chains, and the parallel arrangement of two aromatic rings creates tighter packing with better geometric fit. Cys–Cys residue contacts and the contacts between residues with opposite charges are more frequent in PPI sites [39]. Besides, protein interface regions are less flexible [40] and demonstrate higher sequence conservation rates [38, 41] than other non-binding regions. Conserved interfaces are critical for the maintenance of PPIs throughout evolution.

There are also differences among the interfaces of the various types of PPIs [2]. Depending on the interaction type and its function, the properties that characterise interfaces can vary a lot. For instance, various classes of PPIs differ on the interface propensities of residues [42]. Interfaces of homodimers (complexes made of identical protein chains) are rich in nonpolar and aromatic residues while depleted in polar and charged residues [43], except for Arg which is not excluded in spite of its charge [44]. Interfaces of permanent complexes (i.e. complexes where the constituent proteins remain irreversibly bound after the initial interaction) are more hydrophobic if compared to those of transient complexes (the two proteins can associate and dissociate during their lifetime) [45]. Proteins forming transient complexes should be stable on their own, thus their interfaces are less hydrophobic. The interfaces of obligate complexes (i.e. stable complexes whose constituent proteins do not exhibit well-folded structure when apart) present higher sequence conservation rates [46] and are more hydrophobic [47] than transient complexes. Salt-bridges and hydrogen bonds occur more frequently in the interfaces of transient complexes [2] while covalent disulphide bridges are quite rare, as they can be found in a few, relatively small, permanent complexes [48].

Proteins belonging to the same functional category recognize their interacting partners by certain types of molecular interactions that are specific to their protein family and local environments. As a result, proteins can show specific binding interactions according to their functional classes of PPI interfaces. In [49], basic differences between homodimeric, heterodimeric, protein–antibody and enzyme–inhibitor protein complexes are explored. Cho et al. [50] showed that three functional classes of transient complexes could be distinguished by only four interaction types (NH ⋯ NH, ion–ion, amine–cation and C^*α*^ − H ⋯ O = C). Moreover, C^*α*^ − H ⋯ O = C interactions were found to be predominant in protease–inhibitor interfaces while ion–ion interactions were found to be specific to signal transduction complexes. In [51], six types of PPI interfaces were studied and significant differences were found in their residue composition and their residue–residue contact preferences, in the interactions between permanent and transient interfaces, and between interactions associating homo-oligomers and hetero-oligomers. Antibody–antigen complexes were found to exhibit quite peculiar binding mechanism, as they do not undergo correlated mutations (the antibody adapts to bind a particular antigen) and their amino acid contact propensities are quite different from those of other protein complexes [52].

Although significant research has been done in the area of protein–protein interactions, the problem of PPI interface prediction is still not fully understood [23]. The selection of an optimal set of biological and physico-chemical features characterising the protein surface is one of the main unresolved issues. There are no known features which can singularly distinguish between interface and non-interface regions of the protein surface, and, the complex, non-linear combinations of features required to describe interaction sites can vary widely from one class of PPIs to another. Moreover, protein interface prediction is an imbalanced classification problem, because the the number of interacting residues of a protein is generally much smaller than that of non-interacting ones. Despite these limitations, several computational methods were reported to achieve good performance in the task of interface prediction for specific protein classes. In [53], Gao et. al. predict interface residues in enzymes with a Random Forest classifier employing the maximum relevance minimum redundancy method followed by incremental feature selection. In [54], a genetic algorithms which searches for known interface 3D templates is used to predict enzyme binding sites. In [55], B-cell epitopes (antigen interface) are predicted from the corresponding protein sequence using a combination of two classifiers, a naïve Bayesian and a random forest classifier, through a voting algorithm. Jespersen et. al. predict B-cell epitopes from antigen sequences with a random forest algorithm trained on the interfaces of known antibody–antigen protein complexes [56]. In [57], paratope (antibody interface) prediction is carried by deriving a set of consensus regions from the structural alignment of known sequentially similar antibodies. In [52], antibody-specific statistics are used to annotate residues with a score indicating their likelihood to belong to the antibody paratope.

In view of the above, we decided to perform binding interface prediction on different classes of proteins in order to gain a better understanding of the various PPI interfaces. In this work we introduce a methodology for the binding interface prediction of proteins given their experimentally-solved 3D structures (PDB files), without any knowledge on their possible binding partners. In order to effectively discriminate between interacting sites and non-interacting sites, we used a set of eight high quality amino acid indices (HQIs) of physico-chemical and biochemical properties extracted from AAindex1 dataset and first introduced in [58]. This set of properties has been employed and validated in several recent publications [23, 59–63]. We mapped these HQIs onto the voxelised representation of the protein surface, obtaining a geometrical representation of the latter enriched with the physico-chemical and biochemical properties of the underlying residues. Spherical patches are then uniformly sampled from the protein surface and, for each patch, a rotationally invariant local descriptor based on 3D Zernike moments is computed. The 3D Zernike descriptors (3DZDs) possess several attractive features such as a compact representation, rotational and translational invariance, and have been shown to adequately capture global and local protein surface shape [64–66] and to naturally represent physico-chemical properties on the molecular surface [67]. 3DZDs are employed to quickly evaluate the shape and physico-chemical similarity of local surface patches, since similar patches have similar descriptors. In order to handle the class imbalance between interface and non-interface local surface patches, we used a combination of undersampling of the majority class and oversampling of the minority class. We employed the stability selection method know as Randomized Logistic Regression as a feature selection algorithm on the 3DZDs in order to reduce the overall number of features. The resulting reduced descriptors were then used as samples for a binary classification problem: Support Vector Machines were used as a classifier to distinguish interface local surface patches (surface patches belonging to the protein–protein interaction interface) from non-interface ones. This is the first time that 3D Zernike descriptors of eight HQIs mapped on the corresponding protein surfaces are employed in the prediction of PPI interfaces. The proposed method was tested and validated on 16 classes of proteins obtained from the Protein–Protein Docking Benchmark 5.0, for both their bound and unbound states and compared to other state-of-the-art protein interface predictors.

## Methods

### Protein surface representation

In this work we employed the voxelised representation of the Solvent Excluded surface (SES) [68], which can be defined as follows. If we imagine a probe-sphere of radius equal to the size of the solvent molecule as it rolls over the external atoms of the protein, we can define the SES as the union of two surfaces: the portion of the outer atoms’ surface touched by the probe-sphere while it rolls over them, and the inward-facing surface portions of the probe when it touches two or more atoms. The SES represents a continuous functional surface of the molecule, i.e. the surface that is available to interact with. Voxelised surface representations (also known as dot-surfaces or grid-based representations), although simple, are widely appreciated for their accuracy and applicability in various contexts. A voxel (**vo**lumetric pi**xel**) represents a single, discrete data point on a regular grid in the 3D space, and can contain multiple values in order to represent various properties of a certain portion of space in a simple and effective way.

The voxelised SES of proteins were computed with the region-growing Euclidean distance transform methodology described in our previous works [69, 70] at a resolution of 64 voxels per Å^3^, using a 1.4Å radius for the solvent probe. Patch centres are extracted from each protein surface uniformly and at a minimum separation of 1.8Å, while local surface patches are extracted using a sphere with a 6.0Å radius centred at each patch centre. This ensures that there is plenty overlap among patches with neighbouring centres. The 6.0Å patch radius is a recurring value in many algorithms which employ spherical patches [66, 68, 71–73], because it is an approximation of the radius of an amino acid [71]. The 3D Zernike Descriptors used in this work were computed up to a maximal order of 20, which corresponds a vector of 121 invariants per descriptor. 3DZDs of maximal order 20 have been shown to adequately capture shape complementarity at the protein–protein interface [66].

### Interfacial regions of the protein surface

The recognition of PPI interface regions can be seen as a classification problem, i.e., each local surface patch is assigned to one of the two classes: *interface surface patches*, and *non-interface surface patches*. Consequently, the problem may be solved using statistical and machine learning techniques such as Support Vector Machines. A clear definition of interacting local surface patches is required in order to predict whether a given patch is involved in protein–protein interactions. However, many alternative definitions are being used to define an interaction site based on 3D structural data [74] which can be grouped into two main approaches: (i) inter-atomic distance between non-hydrogen atoms of different protein chains and (ii) change in accessible surface area (ASA) upon complex formation.

In this work, we used the following definition of interface and non-interface local surface patches. Let *P*_1_ and *P*_2_ be two proteins in a given complex whose 3D structure is known, and let *SES*(*P*_1_) and *SES*(*P*_2_) be the corresponding voxelised SES representations. The interface $I_{P_{1}}$ of protein *P*_1_ is defined as the set of voxels from *SES*(*P*_1_) which are within a 4.5Å distance from some heavy atom in *P*_2_, i.e.:

1 $$ \begin{aligned} I_{P_{1}} = \lbrace \boldsymbol{v} \in SES(P_{1}) \mid & \exists\ \text{atom}\ a \in P_{2} \\ &\text{such that}\ d(\boldsymbol{v}, a) \leq 4.5 \text{\r{A}{}} \rbrace~. \end{aligned}  $$

Equivalently, the interface $I_{P_{2}}$ of protein *P*_2_ is defined as:

2 $$ \begin{aligned} I_{P_{2}} = \lbrace \boldsymbol{v} \in SES(P_{2}) \mid & \exists\ \text{atom}\ a \in P_{1} \\ &\text{such that}\ d(\boldsymbol{v}, a) \leq 4.5 \text{\r{A}{}} \rbrace \enspace. \end{aligned}  $$

A patch is an interface patch if at least 80% of its surface voxels are located in the current protein’s interface, otherwise the patch is categorised as a non-interface patch.

### Residue feature set

In order to reliably predict PPI interface residues, the physico-chemical characteristics (features) that can best discriminate between interacting and non-interacting sites must be identified. The choice of such features is critical for the success of a predictor [16]. The AAindex [75] is a database of numerical indices representing various physicochemical and biochemical properties of residues and residue pairs derived from published literature. An amino acid index is a set of 20 numerical values representing any of the different physicochemical and biological properties of each amino acid: the AAindex1 section of the database is a collection of 566 such indices (Release 9.2, February 2017). By using a consensus fuzzy clustering method on all available indices in the AAindex1, Saha et al. [58] identified three high quality subsets (HQIs) of all available indices (544 at the time), namely HQI8, HQI24 and HQI40. In this work we used the features of the HQI8 amino acid index set (see Table 1) which were identified as follows. Using the correlation coefficient between indices as a distance measure, Saha et al. divided all the available indices in the AAindex1 section into 8 clusters: the elements of the HQI8 subset consist of the medoids (centres) of these clusters.

**Table 1:** The HQI8 subset of amino acid indices from the AAindex database

Entry name	Description
BLAM930101	Alpha helix propensity of position 44 in T4 lysozyme [99].
BIOV880101	Information value for accessibility; average fraction 35% [100].
MAXF760101	Normalized frequency of alpha-helix [101].
TSAJ990101	Volumes including the crystallographic waters using the ProtOr [102].
NAKH920108	AA composition of MEM of multi-spanning proteins [103].
CEDJ970104	Composition of amino acids in intracellular proteins (percent) [104].
LIFS790101	Conformational preference for all beta-strands [105].
MIYS990104	Optimized relative partition energies - method C [106].

### 3D Zernike descriptors

The 3D Zernike descriptors (3DZD) were first used as a representation of the protein surface shape in [64], and have since been employed in several tasks such as global protein structure comparison [65], surface property comparison [67], local surface classification [76], binding ligand prediction by pocket-pocket similarity detection [77–79] and pocket-ligand complementarity evaluation [80, 81], and protein-protein docking prediction [66] with quite satisfactory results. 3DZDs present several advantages over other surface representations. For instance, they can represented protein surfaces and the corresponding properties very compactly as a vector of numbers. 3DZDs are invariant to rotations and translations, i.e. they are not affected by the initial orientation of the molecular surface. Because of this property, time-consuming spatial alignments of proteins are not required and the descriptors can be precomputed and stored. The 3DZDs can be computed for any 3D image, and are thus suitable for representing physico-chemical properties on the molecular surface as the electrostatic potential or the hydrophobicity [67]. Lastly, by changing the order of the series expansion, the resolution of the surface representation can be easily controlled.

Each patch of the enriched protein surface is represented by the 3D Zernike descriptors. The 3DZD are a series expansion of a 3D function which exhibit several desirable properties such as compactness of the representation, roto-translational invariance and minimum information redundancy (orthonormality). In what follows we will provide a brief description of the 3DZD. Refer to [82] for the exhaustive mathematical derivation and to [83] for the implementation details. The 3D Zernike functions $Z_{nl}^{m}$ of order *n* and repetition *m* are defined as

3 $$ Z_{nl}^{m}(r, \theta, \phi)=R_{nl}(r) \cdot Y_{l}^{m}(\theta, \phi) \enspace.  $$

$Y_{l}^{m}(\theta, \phi)$ are the spherical harmonics in polar coordinates of *l*^th^ degree, where *l* ≤ *n*, *m*∈ {−*l*,−*l*+1,−*l*+2,…,*l*−1,*l*}, with *n*−*l* an even number. *R*_*nl*_(*r*) are the radial polynomials of radius *r* which guarantee the orthonormality of the $Z_{nl}^{m}(r, \theta, \phi)$ polynomials in Cartesian coordinates. The expression of $Z_{nl}^{m}$ can be rewritten in Cartesian coordinates as a linear combination of monomials of order up to n:

4 $$ Z_{nl}^{m}(\boldsymbol{x}) = \sum\limits_{r+s+t \leq n} \chi_{nlm}^{rst} \cdot x^{r} y^{s} z^{t}~.  $$

The 3D Zernike moments $\Omega _{nl}^{m}$ of function $f(\boldsymbol {x}), \boldsymbol {x}\in \mathbb {R}^{3}$ are defined as:

5 $$ \Omega_{nl}^{m} := \frac{3}{4\pi}\int_{\lvert\boldsymbol{x} \rvert\leq 1} f(\boldsymbol{x})\overline{\boldsymbol{Z}_{nl}^{m}(\boldsymbol{x})}d\boldsymbol{x}~.  $$

Using Eq. 4, the 3D Zernike moments $\Omega _{nl}^{m}$ of an object can be written as a linear combination of geometric moments of order up to n

6 $$ \Omega_{nl}^{m}= \frac{3}{4\pi}\cdot\sum_{r+s+t\leq n}\overline{\chi_{nlm}^{rst}}\cdot M_{rst}~,  $$

where *M*_*rst*_ is the geometric moment of the object scaled to fit in the unit ball

7 $$ M_{rst} = \int_{\lvert\boldsymbol{x} \rvert \leq 1} f(\boldsymbol{x}) \cdot x^{r} y^{s} z^{t} d\boldsymbol{x} \enspace,  $$

where $\boldsymbol {x} \in \mathbb {R}^{3}$ is the vector $\boldsymbol {x} = \left (x, y, z\right)^{\intercal }$.

The 3D Zernike moments $\Omega _{nl}^{m}$ are not invariant under rotations. In order to achieve invariance, moments are collected into (2*l*+1)-dimensional vectors $\boldsymbol {\Omega }_{nl}=\left (\Omega _{nl}^{l}, \Omega _{nl}^{l-1}, \Omega _{nl}^{l-2}, \dots, \Omega _{nl}^{-l}\right)^{\intercal }$, and the rotationally invariant 3D Zernike descriptors *F*_*nl*_ are defined as norms of vectors ***Ω***_*nl*_:

8 $$ F_{nl} := \left\lVert\boldsymbol{\Omega}_{nl}\right\rVert~.  $$

Given the maximum moment order *N*, the number of 3D Zernike descriptors can be easily determined by using the following formula:

9 $$ \text{No. 3DZDs}=\left\{ \begin{array}{ll} \left(\frac{N+2}{2} \right)^{2}, & \text{if \textit{N} is even}\\ \frac{\left(N+1\right)\left(N+3\right)}{4}, & \text{if \textit{N} is odd}~. \end{array} \right.  $$

### Patch representation using 3D Zernike descriptors

The physico-chemical and biochemical properties described in the HQI8 amino acid index set are mapped on the voxelised representation of the protein’s SES. Depending on the amino acid it belongs to, each atom in the protein is assigned the corresponding numeric values of the properties scaled by the atom’s radius. For a given amino acid index, each voxel in the protein’s SES is assigned the corresponding value of the atom occupying that voxel. If a voxel belongs to two or more atoms (i.e. if two or more atoms overlap), then the sum of the corresponding values of the overlapping atoms is assigned to that voxel. If a voxel does not belong to the SES of the current protein, its value is set to zero.

Eight 3D functions are thus defined, each describing one of the properties of the HQI8 set. For a given protein *P*, these functions are formally defined as follows. Let *A*_*P*_ be the set of atoms in the current protein *P*, and let $\Phi _{i}: A_{P} \rightarrow \mathbb {R}$ the function which assigns to each atom the numeric value of the corresponding amino acid for a given amino acid index *i*∈HQI8. Then, for a given amino acid index *i*∈HQI8, the corresponding property is mapped on the *SES*(*P*) according to the following 3D function: 
10

where *r*_*a*_ is the radius of atom *a*, and  is the indicator function for atom *a* defined as: 
11

Zernike descriptors cannot be used to distinguish positive valued functions from negative valued ones (see the Additional file 1 for a concise mathematical justification). For instance, a surface patch with a certain charge distribution pattern would be indistinguishable from another patch with the same shape and inverted electrostatic charges in terms of 3DZDs. This can be avoided by considering a 3D function *f*(***x***) as the difference of its positive part *f*^+^(***x***)= max(*f*(***x***),0) with its negative part *f*^−^(***x***)=− min(*f*(***x***),0), i.e. *f*(***x***)=*f*^+^(***x***)−*f*^−^(***x***), and by computing the 3DZDs of these two functions separately.

Three of the amino acid indices in HQI8 can assume both positive and negative values, namely BLAM930101, BIOV880101 and MIYS990104, while the remaining five indices assume positive values only. The positive and negative parts were considered separately for these three indices, yielding a total of 11 3DZDs describing the HQI8 properties for each local surface patch. The maximal order 20 was used for the calculation of the 3DZDs, thus, according to Eq. 9, each patch is characterised with a total of 11×121=1331 features.

### Support vector machine

Support vector machine (SVM) is a binary classification technique introduced by Vapnik et al. [84–86]. While traditional binary classification methods generally minimize the empirical training error, SVM minimizes the upper bound of the generalization error by maximizing the margin between the separating hyperplane and the data, abiding to the structure risk minimization principle for model selection. Striking feature of SVM is the property of compacting information contained in the training data, and providing a sparse representation even when using a small number of data points.

A binary classification problem usually involves separating data into training and test sets. The instances (samples) of the training set are the pairs (***x***_*i*_,*y*_*i*_), where ***x***_*i*_ is a vector representing the features or attributes of the given sample and *y*_*i*_∈{−1,+1} is the corresponding class label. The goal of SVM is to produce a model based on the training data which predicts the class labels of the test data given only the feature vectors of the test data. This is achieved by solving the following optimisation problem:

12 $$ \begin{aligned} \min_{\boldsymbol{w}, b, \boldsymbol{\xi}} \enspace & \frac{1}{2} \boldsymbol{w}^{\intercal} \boldsymbol{w} + C\sum_{i=1}^{l} \xi_{i} \\ \text{subject to} \enspace & y_{i}\left(\boldsymbol{w}^{\intercal} \phi(\boldsymbol{x}_{i}) + b \right) \geq 1 - \xi_{i}, \\ & \xi_{i} \geq 0, i=1, \dots, l ~, \end{aligned}  $$

where *ϕ*(***x***_*i*_) maps ***x***_*i*_ into a higher-dimensional (and potentially even an infinite-dimensional) space, and *C*>0 is the penalty parameter of the error term. In practice the dual formulation of this problem is solved instead, due to high dimensionality of the vector variable ***w***:

13 $$ \begin{aligned} \min_{\boldsymbol{\alpha}} \enspace & \frac{1}{2} \boldsymbol{\alpha}^{\intercal} y_{i} y_{j} \phi(\boldsymbol{x}_{i})^{\intercal} \phi(\boldsymbol{x}_{j}) \boldsymbol{\alpha} -\boldsymbol{e}^{\intercal} \boldsymbol{\alpha} \\ \text{subject to} \enspace & \boldsymbol{y}^{\intercal} \boldsymbol{\alpha} = 0, \\ & 0 \leq \alpha_{i} \leq C, i=1, \dots, l \enspace, \end{aligned}  $$

where $\boldsymbol {e} = \left [1, 1, \dots, 1 \right ]^{\intercal }$ is the vector of all ones.

After solving the dual problem, the optimal ***w*** is given by

14 $$ \boldsymbol{w}=\sum\limits_{i=1}^{l} y_{i} \alpha_{i}\phi(\boldsymbol{x}_{i}) \enspace,  $$

and by setting $K(\boldsymbol {x}_{i}, \boldsymbol {x}_{j}) = \phi (\boldsymbol {x}_{i})^{\intercal } \phi (\boldsymbol {x}_{j})$, the decision function is given by:

15 $$ \begin{aligned} f(\boldsymbol{x}) &= \text{sgn} \left(\boldsymbol{w}^{\intercal} \phi(\boldsymbol{x}) + b \right) \\ &= \text{sgn} \left(\sum\limits_{i=1}^{l}\ y_{i} \alpha_{i} K(\boldsymbol{x}_{i}, \boldsymbol{x}) + b \right)~. \end{aligned}  $$

Please note that there is no need to compute the mapped feature vectors *ϕ*(***x***) explicitly. Instead, only the dot products between mapped feature vectors are calculated $K(\boldsymbol {x}_{i}, \boldsymbol {x}_{j}) = \phi (\boldsymbol {x}_{i})^{\intercal }\phi (\boldsymbol {x}_{j})$. *K*(***x***_*i*_,***x***_*j*_) is also known as *kernel function*.

SVM can perform non-linear classification in the feature space by finding a separating hyperplane with maximal margin in the higher dimensional space generated by *ϕ*(·). This is easily done by using different kernel functions generating *ϕ*(·). The most used kernels are given in Table 2. Although the performance of SVM mostly depends on the choice of an appropriate kernel function, there is no optimal way to choose an optimal kernel function within a data-driven approach.

**Table 2:** The four basic kernel functions

Kernel name	Mathematical formulation
Linear	$K(\boldsymbol {x}_{i}, \boldsymbol {x}_{j})=\boldsymbol {x}_{i}^{\intercal } \boldsymbol {x}_{j}$
Polynomial	$K(\boldsymbol {x}_{i}, \boldsymbol {x}_{j})=\left (\gamma \boldsymbol {x}_{i}^{\intercal } \boldsymbol {x}_{j} + r\right)^{d}, \gamma > 0$
Radial basis function (RBF)	*K*(***x***_*i*_,***x***_*j*_)=exp(−*γ*∥***x***_*i*_−***x***_*j*_∥^2^),*γ*>0
Sigmoid	$K(\boldsymbol {x}_{i}, \boldsymbol {x}_{j})=\text {tanh}\left (\gamma \boldsymbol {x}_{i}^{\intercal } \boldsymbol {x}_{j} + r\right), \gamma > 0$

*γ*, *r* and *d* are kernel parameters

In this work, interface local patch descriptors are labelled as positive samples (+1) and non-interface ones are labelled as negative samples (−1). Therefore, our interface recognition problem is actually a binary classification problem which can be handled by a SVM. In this work we used the SVM implementation provided in the scikit-learn Python module for machine learning version 0.18.1 [87].

### Performance measures

The PPI interface prediction based on local surface patch descriptors is a binary classification problem, thus, a number of commonly used measures can be employed to evaluate the performance. These methods include accuracy (A), precision (P), recall (R), F_1_ score (F_1_) and the Matthews correlation coefficient (MCC) (see Table 3).

**Table 3:** Performance measures for the binary classification problem: TP – true positives, TN – true negatives, FP – false positives, FN – false negatives

Measure	Mathematical formulation	Comment
Accuracy	A $=\frac {\text {TP}+\text {TN}}{\text {TP}+\text {TN}+\text {FP}+\text {FN}}$	Indicates the fraction of correct predictions over the total: not very significant when dealing with imbalanced data.
Precision	P $=\frac {\text {TP}}{\text {TP}+\text {FP}}$	Indicates the fraction of relevant instances among the retrieved ones.
Recall	R $=\frac {\text {TP}}{\text {TP}+\text {FN}}$	Indicates the fraction of relevant instances that have been retrieved over the total relevant instances.
F_1_ score	F$_{1} = 2 \times \frac {\mathrm {P} \times \mathrm {R}}{\mathrm {P} + \mathrm {R}}$	It is the harmonic mean of precision and recall.
Matthews correlation coefficient	MCC $=\frac {\text {TP}\times \text {TN} - \text {FP}\times \text {FN}}{\sqrt {(\text {TP}+\text {FP})(\text {TP}+\text {FN})(\text {TN}+\text {FP})(\text {TN}+\text {FN})}}$	Returns a value between −1 and +1: +1 represents a perfect prediction, 0 no better than random prediction and −1 indicates total disagreement between prediction and observation.

The Receiver Operating Characteristic (ROC) and the Precision–Recall (PR) curve plots and their Area Under the Curve (AUC) can also be used to assess the quality of a binary classifier. The ROC curve is the most commonly used way to visualize the performance of a binary classifier, and AUC is a very good way to summarize its performance in a single number. In this work, the ROC curve of an SVM classifier is created by plotting the True Positive Rate (the fraction of true positives out of the total predicted positives) against the False Positive Rate (the fraction of false positives out of the total predicted negatives), at various threshold values of the intercept term *b* in Eq. 15. The PR curve is obtained by plotting the precision values against the corresponding recall for all threshold values of *b*.

### Dataset

The Protein–Protein Docking Benchmark 5.0 (DB5) [88] was used as dataset in this work. The benchmark consist of 230 non-redundant, high quality structures of protein–protein complexes along with the unbound structures of their components. Non-redundancy is set at the family level of SCOPe 2.03 [89]: two complexes were considered redundant when the pairs of interacting domains were the same at the SCOPe family level. Antibody–antigen complexes were considered redundant only when the SCOP families of the antigens were identical, and at least 80% of the antigen interface residues were shared between the two complexes. The complexes are divided into 8 different classes: (1) Antibody–Antigen (A), (2) Antigen–Bound Antibody (AB), (3) Enzyme–Inhibitor (EI), (4) Enzyme–Substrate (ES), (5) Enzyme complex with a regulatory or accessory chain (ER), (6) Others, G-protein containing (OG), (7) Others, Receptor containing (OR), and (8) Others, miscellaneous (OX). The complexes are further classified based on the conformational changes upon binding into three classes: (1) rigid-body, (2) medium difficulty and (3) difficult.

In order to assess the predictive capabilities of the proposed method on different protein complex classes, we considered the 8 different classes in the DB5 separately. For each class, we also separated the receptor proteins from the ligand ones, thus obtaining 16 separate datasets. We maintained the separation between classes A and AB, although not being biologically different, in order to be able to evaluate the performance variations due to conformational changes upon binding, as there are no unbound structures available for the receptor proteins in the AB class. For each of the 16 datasets, we further reduced redundancy to a maximum of 90% sequence identity between pairs of different (unbound) proteins with the CD-HIT tool [90, 91]. Each dataset was then randomly split into two disjoint sets: a training set of approximately 60% of the number of complexes and a test set of the remaining ∼ 40% (see Table 4).

**Table 4:** Training and test split for each of the 16 protein classes in the Protein–Protein Docking Benchmark 5.0

Dataset	Training set	Test set
A _*r*_	1AY1.HL (1BGX), 1BVL.BA (1BVK), 2FAT.HL (2FD6), 2I24.N (2I25), 3EO0.AB (3EO1), 3G6A.LH (3G6D), 3HMW.LH (3HMX), 3L7E.LH (3L5W), 3MXV.LH (3MXW), 3V6F.AB (3V6Z), 4GXV.HL (4GXU)	1FGN.LH (1AHW), 1DQQ.CD (1DQJ), 1QBL.HL (1WEJ), 1GIG.LH (2VIS), 2VXU.HL (2VXT), 3RVT.CD (3RVW), 4G5Z.HL (4G6J)
A _*l*_	1TAQ.A (1BGX), 3LZT (1BVK), 1A43 (1E6J), 1YWH.A (2FD6), 1IK0.A (3G6D), 1F45.AB (3HMX), 3M1N.A (3MXW), 3F5V.A (3RVW), 3KXS.F (3V6Z), 1DOL.A (4DN4), 4I1B.A (4G6J), 1RUZ.HIJKLM (4GXU)	1TFH.A (1AHW), 1HRC (1WEJ), 2VIU.ACE (2VIS), 1J0S.A (2VXT), 1QM1.A (2W9E), 1TGJ.AB (3EO1), 3F74.A (3EOA), 2FK0.ABCDEF (4FQI)
AB _*r*_	1BJ1.HL (1BJ1), 1FSK.BC (1FSK), 1I9R.HL (1I9R), 1K4C.AB (1K4C), 1KXQ.H (1KXQ), 2JEL.HL (2JEL), 1QFW.HL (9QFW)	1IQD.AB (1IQD), 1NCA.HL (1NCA), 1NSN.HL (1NSN), 1QFW.IM (1QFW), 2HMI.CD (2HMI)
AB _*l*_	2VPF.GH (1BJ1), 1BV1 (1FSK), 1D7P.M (1IQD), 7NN9 (1NCA), 1HRP.AB (1QFW), 1S6P.AB (2HMI), 1POH (2JEL)	1ALY.ABC (1I9R), 1JVM.ABCD (1K4C), 1PPI (1KXQ), 1KDC (1NSN)
EI _*r*_	1QQU.A (1AVX), 1PIG (1BVN), 1JAE.A (1CLV), 1EAX.A (1EAW), 1TRM.A (1EZU), 4PEP (1F34), 2PKA.XY (1HIA), 1AKL.A (1JIW), 3GMU.B (1JTG), 1QLP.A (1OPH), 1SCD.A (1OYV), 1X9Y.A (1PXV), 2DCY.A (2B42), 966C.A (2J0T), 1ZM8.A (2O3B), 1SUP (2SIC), 1A3S.A (3A4S), 2QA9.E (3SGQ), 3VLA.A (3VLB), 4HWX.AB (4HX3), 1UNK.D (7CEI)	2CGA.B (1ACB), 1RGH.B (1AY7), 1HCL (1BUH), 2TGT (1D6R), 9RSA.B (1DFJ), 9EST.A (1FLE), 1CK7.A (1GXD), 3QI0.A (1JTD), 1J06.B (1MAH), 1UDH. (1UDI), 2GHU.A (1YVB), 1KWM.A (1ZLI), 8CPA.A (4CPA), 1ERK.A (4IZ7)
EI _*l*_	1EGL (1ACB), 1BA7.B (1AVX), 1HOE (1BVN), 1HPT (1CGI), 1QFD.A (1CLV), 1F32.A (1F34), 1PMC.A (1GL1), 1BX8 (1HIA), 1BTL.A (1JTD), 1ZG4.A (1JTG), 1UTQ.A (1OPH), 1PJU.A (1OYV), 1LU0.A (1PPE), 1NYC.A (1PXV), 1B1U.A (1TMQ), 1CEW.I (1YVB), 2JTO.A (1ZLI), 1ZFI.A (2ABZ), 1T6E.X (2B42), 1D2B.A (2J0T), 2NNR.A (2OUL), 2CI2.I (2SNI), 2UUX.A (2UUY), 3A4R.A (3A4S), 3VL8.A (3VLB), 1C7K.A (4HX3)	1A19.B (1AY7), 1DKS.A (1BUH), 1K9B.A (1D6R), 2BNH (1DFJ), 9PTI (1EAW), 1ECZ.AB (1EZU), 2REL.A (1FLE), 1BR9.A (1GXD), 2RN4.A (1JIW), 1FSC (1MAH), 2GKR.I (1R0R), 2UGI.B (1UDI), 1J57.A (2O3B), 3SSI (2SIC), 1H20.A (4CPA), 2LS7.A (4IZ7), 1M08.B (7CEI)
ER _*r*_	1IXM.AB (1F51), 1BU6.O (1GLA), 1AUQ (1M10), 1JXQ.A (1NW9), 1B3K.A (1OC0), 1R6C.X (1R6Q), 2FXS.A (1US7), 2AYN.A (2AYO), 3OWG.A (2GAF), 1L7E.AB (2OOR), 1YZU.A (2OT3), 2YVF.A (2YVJ), 2D1I.A (2Z0E), 2EDI.A (3FN1), 1BPB.A (3K75), 1UPL.A (4FZA)	1AUQ (1IJK), 1JMJ.A (1JMO), 3EED.AB (1JWH), 1JZO.AB (1JZD), 1V8Z.AB (1WDW), 1MH1 (2NZ8), 4JJ7.AB (3H11), 3LVM.AB (3LVK), 3PC6.A (3PC8), 1XVB.ABCDEF (4GAM)
ER _*l*_	1SRR.C (1F51), 1FVU.AB (1IJK), 2OPY.A (1NW9), 2W0G.A (1US7), 1GEQ.A (1WDW), 1VPT.A (2GAF), 1NTY.A (2NZ8), 1E3T.A (2OOR), 1TXU.A (2OT3), 2E4P.A (2YVJ), 1V49.A (2Z0E), 2LQ7.A (3FN1), 1DCJ.A (3LVK), 3PC7.A (3PC8), 3GGF.A (4FZA), 1CKV.A (4GAM)	1F3Z.A (1GLA), 2CN0.HL (1JMO), 3C13.A (1JWH), 1JPE.A (1JZD), 1M0Z.B (1M10), 2JQ8.A (1OC0), 2W9R.A (1R6Q), 2FCN.A (2AYO), 3H13.A (3H11), 3K77.A (3K75)
ES _*r*_	1E1N.A (1E6E), 1GJR.A (1EWY), 1B39.A (1FQ1), 1N0V.C (1ZM4), 3UIU.A (2A1A), 2BBK.JM (2MTA), 1SUR.A (2O8V), 2OOA.A (2OOB), 1GIQ.A (4H03), 4LW2.AB (4LW4)	1CL0.A (1F6M), 1QUP.A (1JK9), 1JB1.ABC (1KKL), 1L6P (1Z5Y), 1U90.A (2A9K), 1J54.A (2IDO), 1CCP (2PCC)
ES _*l*_	1CJE.D (1E6E), 1CZP.A (1EWY), 1FPZ.F (1FQ1), 2JCW.A (1JK9), 2HPR (1KKL), 1Q46.A (2A1A), 2C8B.X (2A9K), 1SE7.A (2IDO), 2RAC.A (2MTA), 1NI7.A (4LW4)	2TIR.A (1F6M), 2B1K.A (1Z5Y), 1XK9.A (1ZM4), 1YJ1.A (2OOB), 1YCC (2PCC), 1IJJ.A (4H03)
OG _*r*_	1QG4.A (1A2K), 1AB8.AB (1AZS), 1CTQ.A (1BKD), 1MH1 (1E96), 1MH1 (1I4D), 5P21.A (1LFD), 6Q21.D (1WQ1), 2ZKM.X (2FJU), 1GFI.A (2GTP), 1MH1 (2H7V), 3CPI.G (3CPH)	1TND.C (1FQJ), 1A4R.A (1GRN), 1MH1 (1HE1), 821P (1HE8), 1RRP.AB (1K5D), 1HUR.A (1R8S), 2BME.A (1Z0K), 1FKM.A (2G77)
OG _*l*_	1OUN.AB (1A2K), 1AZT.A (1AZS), 1HH8.A (1E96), 1RGP (1GRN), 1HE9.A (1HE1), 1OXZ.A (1J2J), 1LXD.A (1LFD), 1R8M.E (1R8S), 1WER (1WQ1), 1YZM.A (1Z0K), 1Z06.A (2G77)	1FQI.A (1FQJ), 1TBG.DH (1GP2), 1A12.A (1I2M), 1F59.A (1IBR), 1YRG.B (1K5D), 2BV1.A (2GTP), 1G16.A (3CPH)
OR _*r*_	1BUY.A (1EER), 1QFK.HL (1FAK), 1B98.AM (1HCF), 1NOB.F (1KAC), 1MKF.AB (1ML0), 1FZV.AB (1RV6), 1BEC (1SBB), 1ACC.A (1T6B), 1U5Y.ABD (1XU1), 1JX6.A (1ZHH), 1YWH.A (2I9B), 3L88.ABC (3L89), 1H0C.AB (3R9A), 1N6U.A (3S9D)	3AVE.AB (1E4K), 1C3D (1GHQ), 1G0Y.R (1IRA), 1MZN.AB (1K74), 1TGK (1KTZ), 1BQU.A (1PVH), 1R42.A (2AJF), 2BBA.A (2HLE), 1S62.A (2X9A)
OR _*l*_	1LY2.A (1GHQ), 1WWB.X (1HCF), 1EMR.A (1PVH), 1QSZ.A (1RV6), 1SHU.X (1T6B), 2HJE.A (1ZHH), 2GHV.E (2AJF), 1IKO.P (2HLE), 2I9A.A (2I9B), 2X9B.A (2X9A), 1CKL.A (3L89), 2C0M.A (3R9A), 1ITF.A (3S9D), 1M1U.A (4M76)	1FNL.A (1E4K), 1ERN.AB (1EER), 1TFH.B (1FAK), 1ILR.1 (1IRA), 1ZGY.AB (1K74), 1F5W.B (1KAC), 1M9Z.A (1KTZ), 1DOL (1ML0), 1SE4 (1SBB), 1XUT.A (1XU1)
OX _*r*_	2CPL (1AK4), 2CLR.DE (1AKJ), 1IJJ.B (1ATN), 1D6O.A (1B6C), 1BDD (1FC2), 3CHY.A (1FFW), 1GRI.B (1GCQ), 1THF.D (1GPW), 1EAN.A (1H9D), 1D4T.AB (1M27), 1IAM.A (1MQ8), 1OFT.AB (1OFU), 1SYQ.A (1RKE), 2PAB.ABCD (1RLB), 1QGV.A (1SYX), 1XQR.A (1XQS), 2FXU.A (1Y64), 1FCH.A (2C0L), 1SZ7.A (2CFH), 2HRA.A (2HRK), 1NG1.A (2J7P), 3CX9.A (2VDB), 3AA7.AB (3AAA), 3BIX.A (3BIW), 1C3D.A (3D5S), 1P97.A (3F1P), 3MYI.A (3H2V), 3KOV.AB (3P57)	1AVV.A (1EFN), 1QRQ.ABCD (1EXB), 1FC1.AB (1FCC), 1QJB.AB (1IB1), 1H15.AB (1KLU), 3MIN.ABCD (1N2C), 1HNF (1QA9), 2F0R.A (1S1Q), 1UCH (1XD3), 1M4Z.A (1ZHI), 1Y20.A (2A5T), 1BIZ.AB (2B4J), 1CRZ.A (2HQS), 3HEC.A (2OZA), 1EQF.A (3AAD), 1Z6R.AB (3BP8), 3BX8.A (3BX7), 3ODQ.AB (3SZK), 1VDD.ABCD (4JCV)
OX _*l*_	4J93.A (1AK4), 3DNI (1ATN), 1CX8.AB (1DE4), 1G83.A (1EFN), 1FC1.AB (1FC2), 2IGG.A (1FCC), 1FWP.A (1FFW), 1GCP.B (1GCQ), 1D0N.B (1H1V), 1STE (1KLU), 1MQ9.A (1MQ8), 2VAW.A (1OFU), 1CCZ.A (1QA9), 3MYI.A (1RKE), 1L2Z.A (1SYX), 1Z1A.A (1ZHI), 1Z9E.A (2B4J), 2BJN.A (2CFH), 1OAP.A (2HQS), 2IYL.D (2J7P), 3FYK.X (2OZA), 1MYO.A (3AAA), 1TEY.A (3AAD), 2R1D.A (3BIW), 2GOM.A (3D5S), 2HD7.A (3DAW), 1WI6.A (3H2V), 3IO2.A (3P57), 2H3K.A (3SZK), 1W3S.A (4JCV)	1CD8.AB (1AKJ), 1IAS.A (1B6C), 1QDV.ABCD (1EXB), 1K9V.F (1GPW), 1ILF.A (1H9D), 1KUY.A (1IB1), 1KW2.B (1KXP), 2NIP.AB (1N2C), 1HBP (1RLB), 1YJ1.A (1S1Q), 1S3X.A (1XQS), 1UX5.A (1Y64), 2A5S.A (2A5T), 1PNE (2BTF), 1C44.A (2C0L), 2HQT.A (2HRK), 2J5Y.A (2VDB), 3BP3.A (3BP8), 3OSK.A (3BX7), 1X0O.A (3F1P)

The table gives the PDB code and chain ID of each protein used in this study (the PDB code in parentheses identifies the corresponding bound complex in the DB5 database)

The interaction interface generally corresponds to a small portion of a protein’s surface, thus, a uniform sampling of the protein surface into local surface patches results in a highly-imbalanced classification problem where the interface patches are the minority class. Most machine learning algorithms do not perform well when the number of instances of one class far exceeds the other, especially when classification accuracy is employed as a figure of merit. This can lead to classifiers that tend to label all the samples as belonging to the majority class, thus trivially obtaining a high accuracy measure.

In this work we used a combination of undersampling of the majority class and oversampling of the minority class in order to balance the training set. The surface of each protein in the training set was first sampled into local surface patches with a minimum separation of 4.5Å between patch centres. Then, only the interface regions were sampled with a minimum separation of 1.0Å between patch centres. This procedure yields more balanced training sets (see Table 5) and guarantees that both the interface and non-interface protein surface regions are sampled in a fairly uniform fashion. We also used the F_1_ score (instead of classification accuracy) as a figure of merit during model evaluation on the training samples. The test samples, on the other hand, were obtained by uniformly sampling the surfaces of the proteins in the test set with a minimum separation of 1.8Å between patch centres, thus retaining the original distribution of positive and negative samples. Table 5 also reports the unbalanced version of the training set obtained with the same parameters.

**Table 5:** The number of interface (positive samples) and non-interface (negative samples) local surface patches in the balanced and unbalanced versions of the training set and in the test set for each protein class

Protein complex class	Receptor ligand	Bound unbound	Balanced training set			Unbalanced training set			Test set		
			Interface patches	Non-interface patches	Total	Interface patches	Non-interface patches	Total	Interface patches	Non-interface patches	Total
A	r	b	4520	5859	10379	1162	31174	32336	629	22141	22770
		u	4545	5867	10412	1169	31070	32239	621	22315	22936
	l	b	4533	6164	10697	1155	32987	34142	674	20382	21056
		u	4809	5926	10735	1224	31529	32753	683	26222	26905
AB	r	b	2207	3806	6013	566	20284	20850	378	18852	19230
		u	2234	3805	6039	580	20181	20761	444	19265	19709
	l	b	2472	2315	4787	633	12329	12962	333	6175	6508
		u	2432	2319	4751	624	12316	12940	332	7877	8209
EI	r	b	8862	7350	16212	2254	39035	41289	1268	28696	29964
		u	7927	7350	15277	2026	38872	40898	1299	29502	30801
	l	b	11291	4172	15463	2890	22222	25112	1541	17243	18784
		u	13344	4170	17514	3397	22154	25551	1471	17085	18556
ER	r	b	5512	7404	12916	1392	39672	41064	1458	37028	38486
		u	5072	7418	12490	1295	39631	40926	1165	36294	37459
	l	b	7615	3779	11394	1953	20199	22152	973	13578	14551
		u	7218	3770	10988	1834	20293	22127	829	13053	13882
ES	r	b	2328	5429	7757	606	29129	29735	486	14498	14984
		u	1821	5361	7182	462	28721	29183	401	14013	14414
	l	b	3004	2231	5235	763	11934	12697	310	7704	8014
		u	2274	2227	4501	574	11866	12440	348	7658	8006
OG	r	b	3960	5557	9517	1008	29796	30804	873	18550	19423
		u	3469	5700	9169	882	30468	31350	764	19318	20082
	l	b	4501	2756	7257	1142	14773	15915	734	14996	15730
		u	3781	2803	6584	988	14887	15875	857	14844	15701
OR	r	b	5109	7344	12453	1298	39365	40663	696	19769	20465
		u	4218	7305	11523	1082	39031	40113	1079	19306	20385
	l	b	4691	3273	7964	1205	17471	18676	1012	14860	15872
		u	4163	3424	7587	1057	18219	19276	1635	14425	16060
OX	r	b	8894	10487	19381	2280	55923	58203	1831	62163	63994
		u	9096	10630	19726	2332	56765	59097	1591	62829	64420
	l	b	10146	8392	18538	2583	44821	47404	2035	32141	34176
		u	9560	9393	18953	2443	50234	52677	2119	33604	35723

### SVM model selection

Choosing an appropriate kernel function with the corresponding best hyper-parameters (which include the penalty *C* and the kernel parameters) is critical for achieving good classification performance with SVMs. Although grid-search is currently the most widely used method for hyper-parameter optimisation in learning algorithms, it can be prohibitively time-consuming since not all hyper-parameters are equally important to tune. Grid search experiments might end up allocating too many trials to the exploration of dimensions with low impact on the final performance and suffer from poor coverage of the more important ones. On the other hand, randomised search experiments were recently proven more efficient in several learning algorithms and datasets [92], and have thus been gaining popularity in several applications.

Feature selection was also performed (on the training samples only) in order to reduce the number of features to a subset of relevant ones, since its benefits are manifold (model simplification, shorter training times, better generalisation and avoiding curse of dimensionality) [93]. In this work, we employed a relatively novel feature selection procedure know as Randomized Logistic Regression [94]. This method works by sub-sampling the training data and fitting a L1-regularised Logistic Regression model where the penalty of a random subset of coefficients has been scaled. By performing this double randomization several times, the method assigns high scores to features that are repeatedly selected across randomizations (see the Additional file 1 for a more detailed description of the feature selection algorithm).

After the feature selection, we performed a randomized search over the hyper-parameters for each of the kernel functions described in Table 2: each parameter was sampled from either a distribution over possible values or a list of discrete choices. The penalty parameter *C* was sampled from the continuous exponential distribution with mean 2000 for all kernel functions. The *γ* parameter was sampled from the continuous exponential distribution with mean 0.01 for the polynomial, RBF and sigmoid kernel functions. The degree *d* parameter of the polynomial kernel was sampled from the discrete uniform distribution $\mathcal {U}\lbrace 2, 10 \rbrace $ (the polynomial kernel of degree 1 is actually the linear kernel), while the *r* parameter of the polynomial and sigmoid kernels was sampled from the continuous uniform distribution $\mathcal {U}\left (-2, 2 \right)$. The computation budget, i.e. the total number of sampled candidates or sampling iterations, was set to 200 iterations for each kernel function.

The hyper-parameter evaluation was carried out through leave-one-out cross-validation (LOOCV) at the protein level. If the training set consists of *k* proteins, in turn, each protein is removed from the training set, and a model is trained on the samples of the remaining *k*−1 proteins. The resulting model is then validated on the samples of the protein that was left out. The performance measure reported by LOOCV is then the average of the values computed in the loop. We used the F_1_ score as a performance measure throughout all experiments.

### Interface residue prediction

In order to predict the set of interface residues in a target protein the predicted interface surface patches must be mapped on the underlying residues. The mapping procedure can be summarized as follows. Each residue in the query protein is assigned an initial score of 0. Then, for each predicted interface surface patch we identify the set of its underlying residues, that is, all the residues with at least one atom within a 6Å distance from the patch centre. The score of each underlying residue is incremented by 1/(1+*d*), where *d* is the minimum distance from its atoms to the current patch centre. At the end of the procedure, each residue in the query protein will be assigned a score which indicates its likelihood of belonging to the PPI interface. Each residue can then be classified as interacting or non-interacting by thresholding on this score.

## Results and discussion

### Model selection results

Table 6 summarises the results of the feature selection procedure with the Randomized Logistic Regression algorithm, describing the number of selected features for each amino acid index, while Table 7 describes the best model chosen by the Randomized Search with leave-one-out cross-validation procedure (see Additional file 2 for the indices of the selected features for each protein class). A relatively small portion of the overall number of features (1331) are extracted for each protein class. This is probably due to the fact that we are mapping residue-wise properties on the molecular surface, and the resulting patterns that arise on the local surface patches are relatively simple. This means that only a few terms of the 3DZDs of order 20 (121-dimensional vectors) are required in order to capture such patterns, and thus distinguish between interface and non-interface surface patches.

**Table 6:** The number of selected features belonging to each physico-chemical property and for each protein class. The + and − signs indicate, respectively, the descriptors of the positive and negative parts of the corresponding amino acid index

Protein complex class	Receptor ligand	Bound unbound	Number of selected features											
			Total	BLAM930101 +	BLAM930101 −	BIOV880101 +	BIOV880101 −	MAXF760101	TSAJ990101	NAKH920108	CEDJ970104	LIFS790101	MIYS990104 +	MIYS990104 −
A	r	b	109	6	9	0	0	13	11	24	2	0	14	30
		u	96	9	9	6	0	4	13	20	3	2	11	19
	l	b	89	5	11	1	4	7	21	5	21	7	5	2
		u	85	8	7	2	6	10	21	3	18	4	4	2
AB	r	b	117	28	0	0	8	4	10	17	20	0	1	29
		u	108	26	0	0	8	3	11	13	16	0	0	31
	l	b	78	9	8	3	7	7	6	3	19	5	5	6
		u	75	13	12	7	6	5	6	1	9	5	8	3
EI	r	b	105	2	0	0	19	11	31	14	10	6	5	7
		u	129	10	12	1	24	8	30	14	11	10	3	6
	l	b	91	6	2	0	4	15	21	1	4	6	17	15
		u	80	4	3	0	5	11	12	5	11	3	3	23
ER	r	b	115	7	0	1	5	23	12	1	23	6	12	25
		u	126	11	1	1	2	3	14	1	27	25	20	21
	l	b	100	6	8	10	14	24	4	1	8	3	14	8
		u	100	9	5	12	10	10	18	3	10	7	8	8
ES	r	b	84	14	0	0	8	1	10	2	18	7	4	20
		u	79	4	4	7	13	5	20	6	8	3	1	8
	l	b	83	0	0	9	10	15	7	0	5	15	15	7
		u	86	11	5	14	7	3	13	4	10	7	12	0
OG	r	b	102	6	9	4	5	29	7	1	8	12	18	3
		u	107	11	13	2	5	23	16	6	11	9	10	1
	l	b	92	8	3	6	4	19	7	8	4	8	7	18
		u	78	10	3	2	1	8	9	7	8	12	5	13
OR	r	b	97	14	0	1	4	23	12	1	5	13	7	17
		u	68	11	0	3	6	5	9	1	18	4	0	11
	l	b	79	3	0	0	2	14	6	1	17	17	17	2
		u	100	11	9	0	8	19	8	3	9	10	7	16
OX	r	b	141	7	14	1	1	9	22	19	34	15	11	8
		u	122	19	4	2	8	12	12	9	24	10	6	16
	l	b	132	19	9	2	14	27	12	3	10	10	15	11
		u	118	13	15	1	12	10	17	2	18	8	10	12
Generic model		b	83	7	0	0	8	8	9	0	15	5	13	18
		u	76	3	8	1	5	10	10	2	12	4	6	15

**Table 7:** The selected (best) SVM model for each protein class, i.e. the penalty *C*, the kernel function and its parameters (*γ*, *d*, *r*)

Protein complex class	Receptor ligand	Bound unbound	No. features	kernel function	*C*	*γ*	*d*	*r*
A	r	b	109	sigmoid	495.33	0.00054	N/A	1.44470
		u	96	rbf	1365.14	0.00039	N/A	N/A
	l	b	89	linear	46.05	N/A	N/A	N/A
		u	85	linear	221.64	N/A	N/A	N/A
AB	r	b	117	poly	23.87	0.03006	2	1.73464
		u	108	poly	426.47	0.01110	3	0.01539
	l	b	78	poly	2157.88	0.01906	7	0.17614
		u	75	poly	4362.45	0.03470	10	-0.03613
EI	r	b	105	poly	1514.50	0.00003	3	-0.15922
		u	129	sigmoid	33.32	0.00029	N/A	-1.61953
	l	b	91	sigmoid	213.15	0.00065	N/A	0.47294
		u	80	poly	1916.02	0.01531	4	0.13840
ER	r	b	115	rbf	9.22	0.00366	N/A	N/A
		u	126	rbf	298.47	0.00222	N/A	N/A
	l	b	100	sigmoid	157.32	0.00024	N/A	-0.24272
		u	100	poly	1001.44	0.00597	5	0.00039
ES	r	b	84	linear	196.85	N/A	N/A	N/A
		u	79	linear	7010.36	N/A	N/A	N/A
	l	b	83	poly	954.76	0.00581	6	1.00104
		u	86	poly	721.43	0.02692	6	0.00022
OG	r	b	102	poly	8543.28	0.01682	6	0.00004
		u	107	rbf	12.42	0.00062	N/A	N/A
	l	b	92	poly	257.51	0.00575	3	-0.00191
		u	78	poly	3421.90	0.01659	8	0.00014
OR	r	b	97	linear	281.56	N/A	N/A	N/A
		u	68	linear	1804.59	N/A	N/A	N/A
	l	b	79	poly	5502.26	0.01908	9	0.00113
		u	100	sigmoid	63.94	0.00261	N/A	-1.90377
OX	r	b	141	rbf	60.29	0.00029	N/A	N/A
		u	122	rbf	747.39	0.00006	N/A	N/A
	l	b	132	poly	383.62	0.02146	8	0.04259
		u	118	poly	779.96	0.02933	9	0.05214
Generic model		b	83	sigmoid	148.639	0.02312	N/A	-1.44779
		u	76	sigmoid	3218.238	0.00196	N/A	1.92731

The “No. features” column indicates the number of selected features resulting from the Randomized Logistic Regression algorithm

It is also worth noticing that the number of selected features of a given amino acid index varies from one protein class to another. For instance, 24 features are selected for the NAKH920108 amino acid index property for the bound version of protein class A _*r*_, while, for the bound version of protein class ES _*l*_ the algorithm selects no features at all for the same amino acid index property. This is consistent with the hypothesis that interfaces of proteins belonging to different classes and carrying different functions can vary widely. Moreover, the number of selected features for a given amino acid index can be used to measure its importance in the characterisation of the PPI interface in a given protein class. The Randomized Logistic Regression algorithm only selects important features which correlate with the classification labels: if few features of an amino acid index property are selected in a given protein class, it means that the given property is not important in discriminating interface from non-interface surface regions for the current class. On the other hand, since the classification labels depend on the selected features, key properties which drive protein interactions in the current protein class will have many of their features selected by the algorithm.

### Prediction results on the test set

**Fig. 1:**
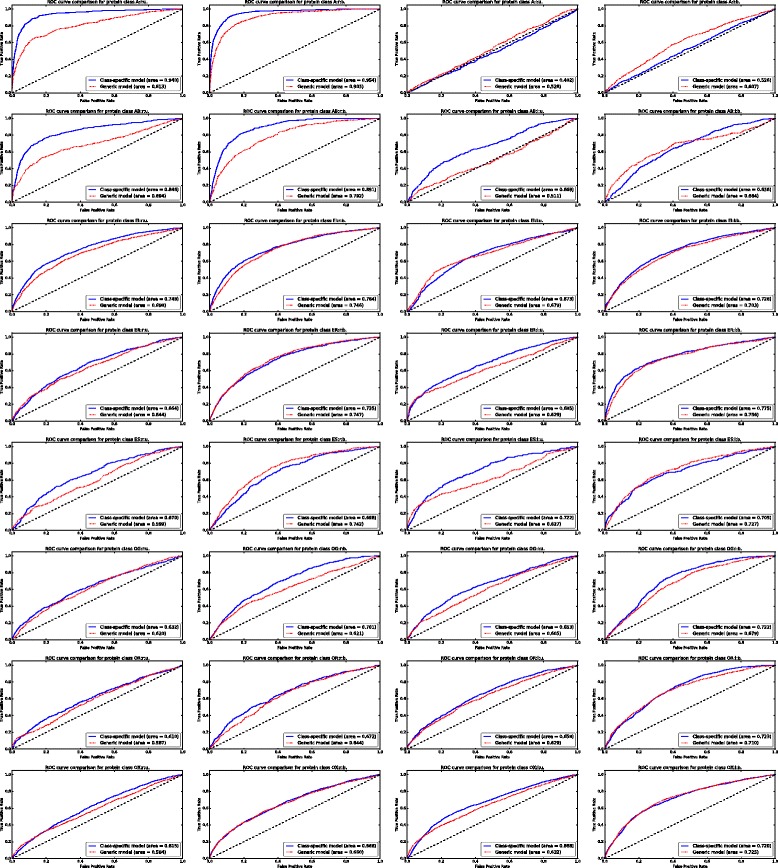
Average Receiver Operating Characteristic curve comparison of the class-specific and generic predictors at the local surface patch level, for each protein class

The performance results for the proposed methodology at the surface patch level on the test set are presented in Table 8 (see Additional file 3 for the prediction results on the test set for each protein). Figure 1 describes the Receiver Operating Characteristic curve for each protein class. The performance of the proposed methodology varies widely from one protein class to another: from a very high AUC-ROC of 94% for class A _*r*_ (95.4% in the bound case) to a much less satisfactory prediction for class A _*l*_ (in both bound and unbound cases). The effect of the conformational changes proteins undergo upon binding can be observed in the differences between the obtained performance values for the bound and unbound versions of the protein classes: for most protein classes, the bound versions obtain better prediction results than the unbound ones.

**Table 8:** Mean and standard deviation (in parentheses) measures of F_1_ score, classification accuracy, precision, recall, MCC and ROC-AUC obtained on the ***test set*** at the local surface patch level using the corresponding best SVM model

Protein complex	Receptor	Bound	F_1_ score	Accuracy	Precision	Recall	MCC	ROC-AUC
class	ligand	unbound						
A	r	b	0.272 (0.101)	0.862 (0.033)	0.166 (0.073)	0.917 (0.056)	0.346 (0.346)	0.954 (0.019)
		u	0.274 (0.121)	0.876 (0.026)	0.169 (0.086)	0.883 (0.084)	0.341 (0.341)	0.939 (0.044)
	l	b	0.093 (0.096)	0.811 (0.045)	0.067 (0.071)	0.182 (0.152)	0.019 (0.019)	0.538 (0.154)
		u	0.097 (0.055)	0.059 (0.030)	0.052 (0.030)	0.987 (0.014)	-0.016 (-0.016)	0.473 (0.053)
AB	r	b	0.230 (0.104)	0.910 (0.023)	0.161 (0.080)	0.590 (0.176)	0.256 (0.256)	0.890 (0.032)
		u	0.228 (0.116)	0.913 (0.020)	0.156 (0.073)	0.546 (0.284)	0.250 (0.250)	0.845 (0.112)
	l	b	0.183 (0.101)	0.653 (0.170)	0.112 (0.069)	0.553 (0.139)	0.110 (0.110)	0.655 (0.119)
		u	0.115 (0.083)	0.246 (0.093)	0.063 (0.049)	0.931 (0.086)	0.071 (0.071)	0.667 (0.180)
EI	r	b	0.156 (0.073)	0.604 (0.084)	0.089 (0.044)	0.770 (0.214)	0.158 (0.158)	0.764 (0.130)
		u	0.148 (0.070)	0.645 (0.070)	0.087 (0.045)	0.705 (0.243)	0.146 (0.146)	0.747 (0.137)
	l	b	0.253 (0.101)	0.535 (0.119)	0.154 (0.068)	0.793 (0.233)	0.167 (0.167)	0.725 (0.177)
		u	0.203 (0.104)	0.360 (0.095)	0.118 (0.065)	0.865 (0.192)	0.086 (0.086)	0.673 (0.150)
ER	r	b	0.145 (0.077)	0.733 (0.043)	0.089 (0.060)	0.580 (0.186)	0.136 (0.136)	0.734 (0.096)
		u	0.109 (0.063)	0.747 (0.055)	0.065 (0.044)	0.465 (0.163)	0.092 (0.092)	0.663 (0.092)
	l	b	0.214 (0.151)	0.494 (0.102)	0.136 (0.127)	0.851 (0.145)	0.167 (0.167)	0.774 (0.147)
		u	0.137 (0.101)	0.087 (0.063)	0.077 (0.062)	0.998 (0.005)	0.019 (0.019)	0.685 (0.144)
ES	r	b	0.031 (0.026)	0.954 (0.020)	0.086 (0.088)	0.023 (0.022)	0.023 (0.023)	0.712 (0.077)
		u	0.121 (0.108)	0.861 (0.040)	0.090 (0.087)	0.281 (0.193)	0.096 (0.096)	0.709 (0.153)
	l	b	0.150 (0.070)	0.665 (0.057)	0.087 (0.043)	0.665 (0.193)	0.142 (0.142)	0.703 (0.144)
		u	0.169 (0.110)	0.636 (0.074)	0.102 (0.077)	0.670 (0.163)	0.148 (0.148)	0.720 (0.140)
OG	r	b	0.184 (0.110)	0.704 (0.039)	0.113 (0.072)	0.552 (0.203)	0.145 (0.145)	0.700 (0.078)
		u	0.144 (0.114)	0.805 (0.031)	0.103 (0.097)	0.340 (0.253)	0.100 (0.100)	0.631 (0.158)
	l	b	0.127 (0.031)	0.373 (0.071)	0.069 (0.017)	0.927 (0.080)	0.125 (0.125)	0.722 (0.069)
		u	0.108 (0.034)	0.089 (0.028)	0.058 (0.019)	0.996 (0.007)	0.037 (0.037)	0.653 (0.119)
OR	r	b	0.121 (0.079)	0.662 (0.068)	0.073 (0.053)	0.558 (0.203)	0.093 (0.093)	0.659 (0.109)
		u	0.103 (0.087)	0.115 (0.047)	0.057 (0.052)	0.968 (0.032)	0.024 (0.024)	0.626 (0.162)
	l	b	0.172 (0.099)	0.269 (0.063)	0.098 (0.063)	0.980 (0.026)	0.120 (0.120)	0.723 (0.085)
		u	0.190 (0.100)	0.592 (0.082)	0.153 (0.171)	0.593 (0.183)	0.095 (0.095)	0.658 (0.119)
OX	r	b	0.108 (0.088)	0.677 (0.081)	0.063 (0.057)	0.555 (0.221)	0.089 (0.089)	0.665 (0.154)
		u	0.081 (0.054)	0.670 (0.087)	0.045 (0.032)	0.490 (0.221)	0.056 (0.056)	0.614 (0.146)
	l	b	0.168 (0.070)	0.369 (0.094)	0.095 (0.043)	0.896 (0.109)	0.120 (0.120)	0.720 (0.113)
		u	0.151 (0.066)	0.352 (0.061)	0.085 (0.041)	0.846 (0.092)	0.077 (0.077)	0.668 (0.110)

To investigate the reasons behind the different performance rates achieved for different protein classes, we measured the average pairwise sequence identity for each protein class (see Table 9, we excluded the pairwise sequence identity measures for chains within the same protein). No particular correlation emerges between the classification performance at the patch level and the average pairwise sequence identity of the different protein classes. For instance, the average pairwise sequence identity in the unbound version of protein class A _*l*_ is 41.52%, which is higher than in some other classes. However, we achieve the lowest classification performance in this class. For this reason we conclude that the performance discrepancies are due to the varying capability of the HQI8 index to adequately represent the diverse interaction patterns that characterise PPIs in the different protein classes.

**Table 9:** Average pairwise sequence identity (in %) for each protein class

Protein class	A				AB				EI				ER			
	r		l		r		l		r		l		r		l	
	u	b	u	b	u	b	u	b	u	b	u	b	u	b	u	b
Whole set	44.04	44.02	41.52	42.32	42.98	42.95	38.19	38.57	41.80	40.86	42.36	43.96	33.70	35.43	41.84	40.48
Training set	47.02	46.88	34.44	40.12	45.15	45.33	41.93	41.93	39.91	39.40	42.75	43.02	33.07	36.28	43.93	42.61
Test set	46.49	46.40	44.43	53.07	39.41	39.41	31.43	43.17	38.98	39.70	49.09	50.80	36.65	36.20	43.98	43.62
Protein class	ES				OG				OR				OX			
	r		l		r		l		r		l		r		l	
	u	b	u	b	u	b	u	b	u	b	u	b	u	b	u	b
Whole set	41.61	40.21	42.31	44.60	34.63	37.31	38.57	37.26	36.60	35.83	37.91	37.71	38.48	39.98	38.61	35.68
Training set	41.52	42.25	46.96	47.31	37.15	33.91	36.34	34.19	37.98	37.30	39.07	38.82	40.32	39.99	39.25	35.24
Test set	35.79	37.69	42.30	47.33	35.41	37.05	44.49	41.81	40.82	45.26	31.76	30.76	37.20	37.61	37.95	39.09

In order to further demonstrate the necessity of developing class-specific protein interface predictors, we trained a generic SVM model based on all the structures in the training set, only differentiating between bound and unbound structures, and evaluated its performance on the test set structures for each protein class. The comparison of the average ROC curves of the class-specific and generic models are given in Fig. 1 for each protein class. In general, the class-specific models obtain better classification performance in terms of ROC-AUC, especially for the bound versions of protein classes A _*r*_, AB _*r*_, OG _*r*_ and the unbound versions of protein classes A _*r*_, AB _*r*_, AB _*l*_, EI _*r*_, ER _*l*_, ES _*r*_, ES _*l*_. Interestingly enough, the class-specific and generic models both obtain very similar results in classes OG, OR and OX (except for the bound version of OG _*r*_). These are the most generic classes in DB5 (i.e. Others, G-protein containing (OG), Others, Receptor containing (OR) and Others, miscellaneous (OX)), thus the benefits of using a class-specific training set are less evident.

### Post-processing

By analysing the results in Table 8 we noticed that for some protein classes the prediction performance in terms of ROC-AUC and recall was high while the other prediction metrics were low. This is due to the fact that the default threshold (*t*=0) used by the SVM classifier (on the *b* term in Eq. 15) does not yield optimal binary classification results, since the employed training set is balanced and does not reflect the natural distribution of interface and non-interface patches. We selected the best threshold value that maximises the average F_1_ score on the training set proteins for each protein class: we used the unbalanced version of the training set for each protein class for this task. The best SVM threshold values obtained for each protein class are reported in the Additional file 1.

Interface regions usually consist of continuous portions of the protein surface. For this reason, the spatial relations among the predicted interface patch centres can be exploited in order to reduce the number of false positive local surface patches. This can be achieved by retaining predicted surface patches which form continuous clusters on the protein surface while discarding the spatially isolated ones. The Isolation Forest (IF) algorithm for outlier detection [95] was used to reduce the number of spatially-isolated false positive local surface patches. Interface regions are composed of contiguous surface patches, thus isolated patches marked as positive by the SVM classifier can be safely discarded. For each query protein, an IF classifier is trained on the coordinates of the LSPs identified as interface patches by the SVM classifier, using their distances from the separating hyperplane as weights. Then, the IF classifier is used on the whole set of surface patches of the query protein to identify the ones belonging to the PPI interface. A contamination parameter must be provided to the IF algorithm: we identified the optimal parameter values for each protein class by testing all contamination values from 0.00 to 0.5 with a constant increment of 0.01, and selected the ones that yielded the best average F_1_ score on the training set of the corresponding protein class. Because the IF for outlier detection is a random algorithm, the F_1_ score was averaged over 100 runs for each contamination value. When the best average F_1_ score was obtained for a contamination value equal to zero we skipped the IF step. The best contamination values obtained for each protein class are reported in the Additional file 1.

### Comparison with other methods

Homology-based (or template-based) approaches constitute the best performing PPI interface prediction methods to date (given the availability of close homologous structures) [16]. These methods infer the biological properties of a query protein from its homologs based on the assumption that homologs share significant similarity in sequence, structure and functional sites. For this reason, in order to assess the prediction capabilities of the proposed methodology, we compared it with two state-of-the-art homology-based PPI interface prediction algorithms: NPS-HomPPI [96] and PrISE [97], and with the well-known structure-based approach SPPIDER [30, 74]. NPS-HomPPI infers interfacial residues for a query protein from the interfacial residues of its homologs. Based on interface conservation thresholds derived from a systematic interface conservation analysis, NPS-HomPPI classifies the templates into either Safe, Twilight or Dark Zone, and uses multiple templates from the best available zone to infer interfaces for query proteins. PrISE is a family of local structural similarity-based computational methods for predicting PPI interface residues. For each target residue in a query protein structure, the spatial neighbours of the target are extracted and represented by their atomic composition and accessible surface areas. PrISE then searches its pre-calculated database for similar structural elements with experimentally determined interface information, and weights them according to their similarity with the structural element of the query protein. SPPIDER is a consensus method that combines the output of 10 Neural Networks using the majority voting. It uses the difference between the predicted and the actual rASA in an unbound structure of a residue as a feature (fingerprint) to predict interfaces.

The assessment was carried out on the structures of the test set described in Table 4, and the performance evaluation was done separately for each protein class. We used the following common definition of the PPI interface for all methods: a residue is considered as interfacial if at least one of its heavy atoms is within a 5Å distance from any other heavy atom of the interacting protein. When possible (i.e. for NPS-HomPPI and PrISE), the interface definition parameter was set accordingly. In the homology-based methods, all homologous structures with sequence identity with the query protein of 90% or above were not considered. We also required the predictions to be expressed as scores or probabilities estimating the likelihood of a residue being in the interface. The default settings were used for all the remaining parameters.

**Fig. 2:**
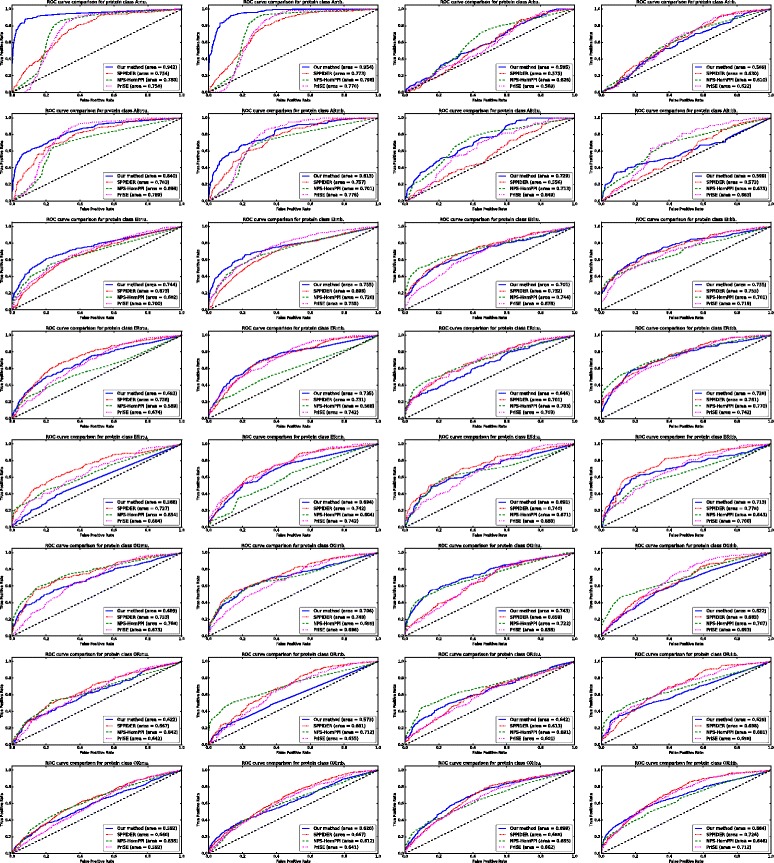
Average Receiver Operating Characteristic curve comparison of the proposed PPI interface prediction method, NPS-HomPPI, PrISE and SPPIDER at the residue level, for each protein class

**Fig. 3:**
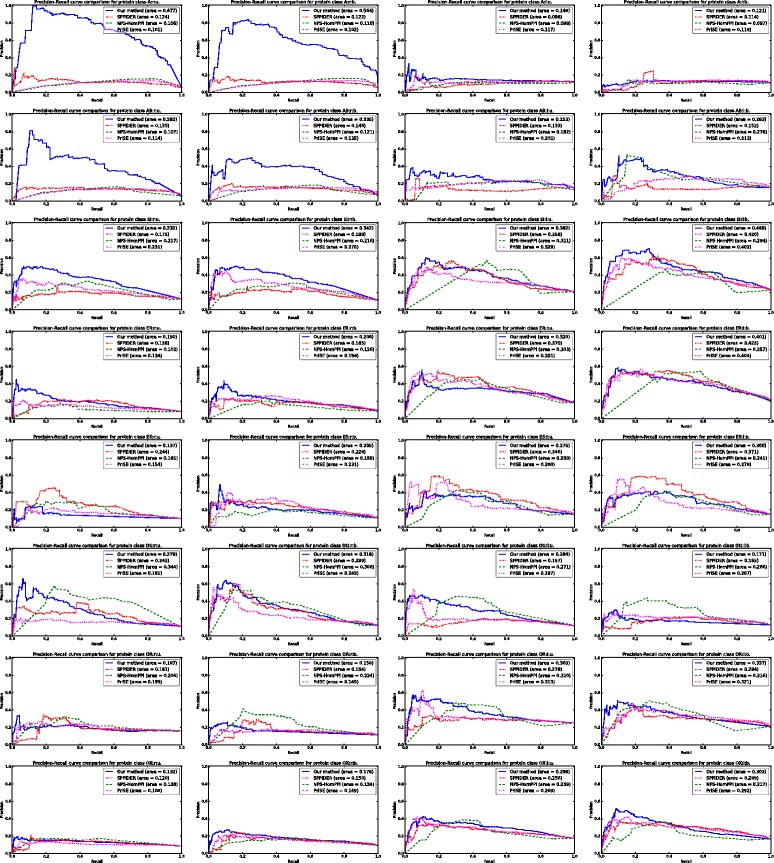
Average Precision–Recall curve comparison of the proposed PPI interface prediction method, NPS-HomPPI, PrISE and SPPIDER at the residue level, for each protein class

By thresholding on the residue score values we computed the average ROC curves and average Precision-Recall curves for each method which are shown in Figs. 2 and 3 respectively. The proposed methodology outperforms the competitor predictors in both the bound and unbound versions of protein classes A _*r*_ and AB _*r*_: the ROC-AUC and PR-AUC values obtained by our predictor are significantly higher than the others. In the unbound version of protein class A _*r*_, our method achieves a ROC-AUC of 94.2% and a PR-AUC of 67.7% while, for the competitors, the maximum ROC-AUC is 78.0% (for NPS-HomPPI) and the maximum PR-AUC is 12.4% (for SPPIDER). Similarly, in the bound version of protein class A _*r*_, our method achieves a ROC-AUC of 95.4% and a PR-AUC of 56.4% while, for the competitors, the maximum ROC-AUC is 79.6% (for NPS-HomPPI) and the maximum PR-AUC is 12.2% (for SPPIDER). In the unbound version of AB _*r*_, the proposed method achieves a ROC-AUC of 84.0% and a PR-AUC of 39.2% while the maximum ROC-AUC for the competitors is 78.9% (for PrISE) and the maximum PR-AUC is 13.5% (for SPPIDER). For the bound version of AB _*r*_ our method obtains a ROC-AUC score of 81.3% and a PR-AUC of 33.5%. The best ROC-AUC obtained by the competitors in the same class is 77.6% (for PrISE) and the best PR-AUC is 14.6% (for SPPIDER). Noticeably better prediction performance is also achieved in the unbound and bound versions of class EI _*r*_: the achieved ROC-AUC values are 74.4% for the unbound and 75.5% for the bound version, while the achieved PR-AUC values are 33.2% for the unbound and 34.2% for the bound version. Although PrISE obtained the same ROC-AUC in the bound version of EI _*r*_, the corresponding PR-AUC is only 27.0%. Slightly better than average prediction results were also obtained in the unbound versions of classes AB _*l*_, OG _*l*_, OX _*l*_ and in the bound version of class EI _*l*_. Our prediction method underperformed compared to the competitors in the unbound versions of classes ES _*r*_, ER _*l*_ and in the bound versions of classes OR _*r*_, OR _*l*_, OG _*l*_. In all other protein classes the prediction capabilities of the proposed methodology followed the average trend of the competitor methods.

The obtained results agree with the initial hypothesis that proteins belonging to different classes exhibit diverse interaction mechanisms. To this end, the choice of a correct set of physico-chemical and biochemical properties characterising the interaction site is crucial, although it might not be possible to identify a comprehensive set of features that works well for all protein classes as the recognition patterns can be very different. Our results suggest that, although a given set of features can effectively discriminate between interface and non-interface surface regions for a given protein class, it can perform very poorly when used on other protein classes. Interface prediction could be further improved by better feature representation and selection methods that can effectively capture complex protein recognition patterns in diverse types of interactions, however, different protein classes should be treated separately.

The HQI8 amino acid index set of physico-chemical and biochemical properties showed very good discriminative capabilities for the interface recognition of some protein classes (A _*r*_, AB _*r*_) while under-performing in others (A _*l*_). Other sets of properties could exhibit better discriminative power in the protein classes where we obtained low prediction performances. Ideally, an optimal set of features could be selected for each protein class in order to correctly identify the class-specific PPI patterns. The proposed methodology can be easily extended to other sets of amino acid properties, which can be similarly mapped on the voxelised protein surface and represented by 3DZDs.

Although not considered in this work, binding partner specificity has recently been reported to greatly affect the quality of predicted PPI interfaces [16], especially in transient protein interactions [96]. Partner-specific interface prediction methods have been shown to outperform several state-of-the-art non-partner-specific ones [22, 34, 98], and apparently, specific interacting partners should be considered in order to reliably predict interface regions. The methodology introduced in this work could be extended in order to predict pairs of interacting local surface patches by feeding the SVM classifier with the concatenation of the corresponding descriptors. However, class imbalance should be handled very carefully, as the number of negative samples (non-interacting patch pairs) would significantly increase with respect to the non-parter-specific case, while the number of positive samples (interacting patch pairs) would roughly remain the same as in the previous case.

## Conclusions

Existing structure-based PPI interface predictors employ 3D structural information to encode statistical properties of surface patches as input feature vectors for binary classifiers while information about the spatial arrangement of atoms and residues is usually ignored. In this study we introduced a novel method for the prediction of PPI interface regions based on 3D Zernike descriptors, HQI8 amino acid index set and SVMs. We demonstrated that 3D Zernike Descriptors of physico-chemical and biochemical amino acid properties mapped on local patches of the protein surface can be used to characterise the latter in order to distinguish between interface and non-interface regions. The 3DZDs are able to capture the similarity among patterns of physico-chemical and biochemical properties mapped on the protein surface arising from the various spatial arrangements of the underlying residues. It is also worth noticing that this is the first time the physico-chemical and biochemical properties of the HQI8 set were mapped directly onto the 3D representation of the protein surface instead of being used to characterise the protein sequence.

This method was tested on 16 protein classes extracted from the Protein–Protein Docking Benchmark 5.0, on both the bound and unbound versions, and was compared with three other state-of-the-art PPI interface predictors, namely SPPIDER, PrISE and NPS-HomPPI. With a resulting ROC-AUC of, respectively, over 94% and 81%, we obtained very good classification results on protein classes A _*r*_ and AB _*r*_ (i.e. antibodies) and even outperformed the competitors also in terms of precision–recall. These results are very encouraging, thus we are planning to develop a specific antigen-binding interface (also known as paratope) prediction method for antibodies with known structure using the 3D Zernike descriptors and the HQI8 amino acid index set. The field of paratope residue prediction appears to be somewhat underdeveloped, with a general paucity of specific predictors, thus any future development in this direction should be quite promising.

Our results show that the choice of a proper set of features characterising the protein interface is crucial for the interface prediction task, and that the optimal set of features strongly depends on the specific protein class. For further improvement of prediction performance, it is necessary to identify an optimal set of features for each protein class or interaction type. As a future development, we plan to test several sets of features on different protein classes in order to widen the predictive capabilities of the proposed method. Including informations regarding possible binding partners in the prediction procedure is also expected to increase the overall performance, although tackling the resulting class imbalance will not be trivial.

The comparison of the class-specific interface prediction models with a generic one, trained on all training samples regardless of the protein class, also confirmed the hypothesis that interface prediction model development should be carried separately for different protein classes. In a certain way, this is similar to the homology-based interface prediction approach: for a given query protein, its closest homolog proteins with known binding sites are retrieved, and the query’s binding site is determined by comparison with the known structures. However, these methods cannot yield good predictions when adequate homologs are not available. Similarly to homology-based predictors, the proposed method requires the availability of several protein classes in order to reliably predict interface regions. Given the ever increasing number of available high-resolution 3D protein structures in public repositories, we expect that more benchmark sets and databases such as the Protein–Protein Docking Benchmark 5.0 which classify proteins into biologically relevant classes will be available. As a future work, we will expand the proposed methodology to other protein classes in order to increase the coverage of its predicting capabilities to as many proteins as possible. A pre-processing step for the determination of the most adequate protein class will be required for proteins with unknown classes. The predictor will then be made available to users throughout a dedicated web server.

The majority of the available protein interface identification methods make predictions at the residue resolution level. Protein–protein docking algorithms, however, require high-resolution atomic level knowledge in order to correctly predict native binding configurations between interacting proteins. The predictions at the local surface patch level can be readily used to guide protein–protein docking methods by limiting the docking search space to the sole surface patches which were predicted as belonging to the interaction interface. Docking algorithms based on local surface descriptor matching can greatly benefit from the proposed approach since this will sensibly limit the number of candidate patch pairs to be evaluated, thus reducing the conformational search space, and consequently reducing both the number of false positives and the required calculation time.

## Additional files

Additional file 1 Contains additional information on some technical aspects of the research. (PDF 1360 kb)

Additional file 2 Contains the indices of the selected features for each protein class. (CSV 1.36 kb)

Additional file 3 Contains the prediction results on the test set for each protein, summarised in Table 8. (XLSX 67.9 kb)
