# Supplementary material for: Exploring the potential of 3D Zernike descriptors and SVM for protein–protein interface prediction
Source: BMC Bioinformatics. 2018 Feb 6;19:35. doi: 10.1186/s12859-018-2043-3 (PMC5802066; doi:10.1186/s12859-018-2043-3)
Supplement: Supplementary file 1 — Contains additional information on some technical aspects of the research. (PDF 1360 kb) [file 12859_2018_2043_MOESM1_ESM.pdf]

Exploring the potential of 3D Zernike descriptors and SVM for protein–protein interface prediction: **Supplementary material**

Sebastian Daberdaku and Carlo Ferrari

Contents

1 3D Zernike descriptors **1**

2 Feature selection **4**

3 SVM model selection results **5**

4 Post-processing **8**

4.1 Best SVM threshold values 8

4.2 Best contamination values 15

1 3D Zernike descriptors

The 3DZD are a series expansion of a 3D function which exhibit several desirable properties such as compactness of the representation, roto-translational invariance and minimum information redundancy (orthonormality). In what follows we will provide a brief description of the 3DZD. Refer to [1] for the exhaustive mathematical derivation and to [2] for the implementation details. The 3D Zernike functions Z_{nl}^m of order n and repetition m are defined as

$$Z_{nl}^m(r, \theta, \phi) = R_{nl}(r) \cdot Y_l^m(\theta, \phi) \ . \tag{1}$$

$Y_l^m(\theta, \phi)$ are the spherical harmonics in polar coordinates of lth degree, where $l \leq n$, $m \in \{-l, -l+1, -l+2, \ldots, l-1, l\}$, with $n-l$ an even number. $R_{nl}(r)$ are the radial polynomials of radius r which guarantee the orthonormality of the $Z_{nl}^m(r, \theta, \phi)$ polynomials in Cartesian coordinates. The expression of Z_{nl}^m can be rewritten in Cartesian coordinates as:

$$\begin{aligned}
Z_{nl}^m(\boldsymbol{x}) =& c_l^m 2^{-m} \sum_{\nu=0}^{k} q_{kl}^{\nu} \sum_{\alpha=0}^{\nu} \binom{\nu}{\alpha} \sum_{\beta=0}^{\nu-\alpha} \binom{\nu-\alpha}{\beta} \\
& \cdot \sum_{u=0}^{m} (-1)^{m-u} \binom{m}{u} \hat{\imath}^u \sum_{\mu=0}^{\lfloor \frac{l-m}{2} \rfloor} (-1)^{\mu} \\
& \cdot 2^{-2\mu} \binom{l}{\mu} \binom{l-\mu}{m+\mu} \sum_{\nu=0}^{\mu} \binom{\mu}{\nu} \\
& \cdot x^{2(\nu+\alpha)+u} \cdot y^{2(\mu-\nu+\beta)+m-u} \\
& \cdot z^{2(\nu-\alpha-\beta-\mu)+l-m} \ ,
\end{aligned} \tag{2}$$

with $\hat{i} = \sqrt{-1}$ and $2k = n - l$. Substituting $r = 2(\nu + \alpha) + u$, $s = 2(\mu - \nu + \beta) + m - u$, $t = 2(\nu - \alpha - \beta - \mu) + l - m$ and setting

$$\begin{aligned} \chi_{nlm}^{rst} =& c_l^m \cdot 2^{-m} \cdot \sum_{\nu=0}^{k} q_{kl}^{\nu} \cdot \sum_{\alpha=0}^{\nu} \binom{\nu}{\alpha} \sum_{\beta=0}^{\nu-\alpha} \binom{\nu-\alpha}{\beta} \\ & \cdot \sum_{u=0}^{m} (-1)^{m-u} \binom{m}{u} \hat{i}^u \sum_{\mu=0}^{\lfloor \frac{l-m}{2} \rfloor} (-1)^{\mu} \\ & \cdot 2^{-2\mu} \binom{l}{\mu} \binom{l-\mu}{m+\mu} \cdot \sum_{\eta=0}^{\mu} \binom{\mu}{\eta} \ , \end{aligned} \tag{3}$$

Z_{nl}^m can be written in a more compact form as a linear combination of monomials of order up to n

$$Z_{nl}^m(\boldsymbol{x}) = \sum_{r+s+t \leq n} \chi_{nlm}^{rst} \cdot x^r y^s z^t \ . \tag{4}$$

The 3D Zernike moments Ω_{nl}^m of function $f(\boldsymbol{x}), \boldsymbol{x} \in \mathbb{R}^3$ are defined as:

$$\Omega_{nl}^m := \frac{3}{4\pi} \int_{|\boldsymbol{x}| \leq 1} f(\boldsymbol{x}) \overline{\boldsymbol{Z}_{nl}^m(\boldsymbol{x})} d\boldsymbol{x} \ . \tag{5}$$

Using Eq. 4, the 3D Zernike moments Ω_{nl}^m of an object can be written as a linear combination of geometric moments of order up to n

$$\Omega_{nl}^m = \frac{3}{4\pi} \cdot \sum_{r+s+t \leq n} \overline{\chi_{nlm}^{rst}} \cdot M_{rst} \ , \tag{6}$$

where M_{rst} is the geometric moment of the object scaled to fit in the unit ball

$$M_{rst} = \int_{|\boldsymbol{x}| \leq 1} f(\boldsymbol{x}) \cdot x^r y^s z^t d\boldsymbol{x} \ , \tag{7}$$

where $\boldsymbol{x} \in \mathbb{R}^3$ is the vector $\boldsymbol{x} = (x, y, z)^{\mathsf{T}}$. An important fact implied by Eq. 6 is that in order to compute the 3D Zernike functions, we only have to compute the geometric moments instead of evaluating the complex exponential and associated Legendre function of spherical harmonics.

The 3D Zernike moments Ω_{nl}^m are not invariant under rotations. In order to achieve invariance, moments are collected into $(2l+1)$-dimensional vectors $\boldsymbol{\Omega}_{nl} = \left(\Omega_{nl}^l, \Omega_{nl}^{l-1}, \Omega_{nl}^{l-2}, \ldots, \Omega_{nl}^{-l}\right)^{\mathsf{T}}$, and the rotationally invariant 3D Zernike descriptors F_{nl} are defined as norms of vectors $\boldsymbol{\Omega}_{nl}$:

$$F_{nl} := \|\boldsymbol{\Omega}_{nl}\| \ . \tag{8}$$

Given the maximum moment order N, the number of 3D Zernike descriptors can be easily determined by using the following formula:

$$\text{No. 3DZDs} = \begin{cases} \left(\frac{N+2}{2}\right)^2, & \text{if } N \text{ is even} \\ \frac{(N+1)(N+3)}{4}, & \text{if } N \text{ is odd} \ . \end{cases} \tag{9}$$

Claim: *Zernike descriptors cannot be used to distinguish positive valued functions from negative valued ones.*

Proof: Let $f(\boldsymbol{x})$ be a 3D function defined inside the unit ball, the Zernike moment of order n, l, m for the function $-f(\boldsymbol{x})$, with $n \in \mathbb{N}$, $(n-l)$ even and $m \in \{-l, \ldots, l\}$ according to Eq. 5 is:

$$\begin{aligned} \Omega_{nl}^m(-f) &= \frac{3}{4\pi} \int_{|\boldsymbol{x}| \leq 1} -f(\boldsymbol{x}) \overline{\boldsymbol{Z}_{nl}^m(\boldsymbol{x})} d\boldsymbol{x} \\ &= -\frac{3}{4\pi} \int_{|\boldsymbol{x}| \leq 1} f(\boldsymbol{x}) \overline{\boldsymbol{Z}_{nl}^m(\boldsymbol{x})} d\boldsymbol{x} \\ &= -\Omega_{nl}^m(f) \ . \end{aligned} \tag{10}$$

The Zernike invariant of order n, l for $-f(\boldsymbol{x})$ according to Eq. 8 is:

$$\begin{aligned} F_{nl}(-f) := \|-\boldsymbol{\Omega}_{nl}\| &= \sqrt{\sum_{m=-l}^{l} (-\Omega_{nl}^{m}(f))\,\overline{(-\Omega_{nl}^{m}(f))}} \\ &= \|\boldsymbol{\Omega}_{nl}\| = F_{nl}(f) \qquad \square \end{aligned} \tag{11}$$

2 Feature selection

Feature selection was performed in order to reduce the number of features to a subset of relevant ones. The benefits of performing feature selection before model construction are manifold (model simplification, shorter training times, better generalisation and avoiding curse of dimensionality) [3]. In this work, we employed a relatively novel method for feature selection known as stability selection [4]. This method is based on sub-sampling in combination with feature selection algorithms: feature selection is performed several times on various random subsets of the data and with various random subsets of features. Each feature can then be ranked based on how frequently it was chosen during the iterations of the algorithm, as features selected more often are considered good features. Relevant features are expected to have high scores, since they are always selected when possible. Weaker, but still relevant features will also have non-zero scores, since they would be selected when stronger features are not present in the currently selected subset, while irrelevant features would have scores close to zero, since they would never be among selected features.

We employed the stability selection method know as Randomized Logistic Regression. This method works by sub-sampling the training data and fitting a L1-regularised Logistic Regression model where the penalty of a random subset of coefficients has been scaled. By performing this double randomization several times, the method assigns high scores to features that are repeatedly selected across randomizations. More formally, let $T = \{(\boldsymbol{x}_i, y_i), i = 1, 2, \ldots, n\}$ be a given training set of n samples for a binary classification problem, and let $\{(\boldsymbol{x}_i, y_i), i \in I\}$ be a random subset of n_I training samples, where $I \subset \{1, 2, \ldots, n\}$ is the set of the corresponding sample indices. The following L1-regularised Logistic Regression fit is obtained:

$$\begin{aligned}\hat{\boldsymbol{w}}_I = \arg\min_{\boldsymbol{w}} \; & \frac{1}{n_I} \sum_{i \in I} \log\left(1 + \exp\left(-y_i(w_0 + \boldsymbol{x}_i^\intercal \boldsymbol{w})\right)\right) \\ & + \lambda \sum_{j=1}^{p} \frac{|w_j|}{s_j} \;,\end{aligned}$$

where $s_j \in \{s, 1\}$ are the outcomes of independent trials of a fair Bernoulli random variable, $0 < s < 1$ is the scaling factor, $\lambda \in \mathbb{R}^+$ is the regularisation parameter of the Logistic Regression and p is the size of the feature vectors $\boldsymbol{x}_i$, i.e. the total number of features. By repeating this procedure across different random sub-samples and Bernoulli trials, one can count the fraction of times the randomized procedure selected each feature, and use these fractions as scores for feature selection. In this work, we used the implementation of the Randomized Logistic Regression method provided in scikit-learn with the default parameters.

3 SVM model selection results

Table 1 gives the corresponding average prediction results obtained with the cross-validation on the balanced training set in terms of F_1 score, classification accuracy, precision, recall, Matthews correlation coefficient and ROC-AUC. The Additional file 4 contains the LOOCV results on the balanced training set for each protein.

There is an evident discrepancy between the average performance values obtained with the LOOCV on the balanced training set and the ones obtained on the test set, for all protein classes. This is mainly due to the different proportions of positive and negative samples in the training set and test set. To verify this hypothesis we performed a modified leave-one-out cross-validation procedure using the best parameters determined earlier for each training set, described as follows. For each protein in the training set, we computed an unbalanced set of samples with the same procedure used to compute the ones in the test set. Given a training set consisting of k proteins, each protein is, in turn, removed from the training set, and a model is trained on the balanced samples of the remaining $k-1$ proteins. Then, instead of using the remaining balanced samples of the left-out protein, we tested the model on an its unbalanced set of samples which retain the original distribution of positive and negative samples. The results, given in Table 2, confirm our hypothesis as the performance values are similar to the ones obtained on the test set (see Additional file 5 for the LOOCV results on the unbalanced training set for each protein).

Table 1: Mean and standard deviation (in parentheses) measures of F_1 score, classification accuracy, precision, recall, MCC and ROC-AUC at the local surface patch level obtained from the LOOCV procedure on the ***balanced training set*** using the corresponding best SVM model.

**Protein complex class**	**receptor ligand**	**bound un-bound**	**F_1 score**	**accuracy**	**precision**	**recall**	**MCC**	**ROC-AUC**
**A**	r	b	0.813 (0.111)	0.858 (0.066)	0.823 (0.064)	0.827 (0.180)	0.702 (0.702)	0.941 (0.044)
		u	0.810 (0.082)	0.854 (0.055)	0.828 (0.080)	0.817 (0.147)	0.695 (0.695)	0.934 (0.040)
	l	b	0.345 (0.166)	0.551 (0.066)	0.617 (0.191)	0.308 (0.221)	0.138 (0.138)	0.630 (0.049)
		u	0.650 (0.104)	0.560 (0.060)	0.528 (0.127)	0.889 (0.109)	0.141 (0.141)	0.620 (0.060)
**AB**	r	b	0.686 (0.206)	0.799 (0.126)	0.760 (0.156)	0.645 (0.253)	0.553 (0.553)	0.841 (0.168)
		u	0.650 (0.232)	0.783 (0.140)	0.735 (0.192)	0.605 (0.276)	0.512 (0.512)	0.817 (0.196)
	l	b	0.567 (0.130)	0.595 (0.079)	0.641 (0.177)	0.547 (0.142)	0.218 (0.218)	0.655 (0.057)
		u	0.700 (0.100)	0.587 (0.104)	0.569 (0.131)	0.947 (0.040)	0.219 (0.219)	0.649 (0.083)
**EI**	r	b	0.743 (0.093)	0.722 (0.087)	0.740 (0.085)	0.767 (0.153)	0.452 (0.452)	0.796 (0.098)
		u	0.622 (0.220)	0.676 (0.130)	0.653 (0.203)	0.622 (0.266)	0.330 (0.330)	0.715 (0.168)
	l	b	0.858 (0.082)	0.796 (0.097)	0.818 (0.086)	0.908 (0.106)	0.448 (0.448)	0.818 (0.132)
		u	0.848 (0.080)	0.767 (0.100)	0.780 (0.114)	0.951 (0.087)	0.353 (0.353)	0.768 (0.080)
**ER**	r	b	0.606 (0.107)	0.695 (0.072)	0.647 (0.127)	0.584 (0.123)	0.358 (0.358)	0.760 (0.074)
		u	0.565 (0.178)	0.701 (0.095)	0.625 (0.175)	0.542 (0.207)	0.354 (0.354)	0.756 (0.120)
	l	b	0.793 (0.094)	0.734 (0.068)	0.738 (0.124)	0.873 (0.096)	0.393 (0.393)	0.773 (0.070)
		u	0.754 (0.110)	0.618 (0.136)	0.617 (0.137)	0.999 (0.002)	0.063 (0.063)	0.714 (0.123)
**ES**	r	b	0.360 (0.200)	0.638 (0.125)	0.531 (0.237)	0.409 (0.334)	0.188 (0.188)	0.706 (0.088)
		u	0.295 (0.231)	0.625 (0.223)	0.474 (0.353)	0.396 (0.369)	0.202 (0.202)	0.725 (0.090)
	l	b	0.671 (0.143)	0.674 (0.089)	0.721 (0.178)	0.640 (0.130)	0.336 (0.336)	0.730 (0.094)
		u	0.677 (0.164)	0.689 (0.087)	0.653 (0.167)	0.723 (0.174)	0.364 (0.364)	0.733 (0.103)
**OG**	r	b	0.505 (0.178)	0.643 (0.102)	0.583 (0.179)	0.492 (0.230)	0.266 (0.266)	0.712 (0.131)
		u	0.403 (0.108)	0.667 (0.073)	0.586 (0.160)	0.329 (0.114)	0.233 (0.233)	0.693 (0.097)
	l	b	0.757 (0.088)	0.668 (0.090)	0.678 (0.122)	0.885 (0.109)	0.289 (0.289)	0.748 (0.118)
		u	0.710 (0.115)	0.566 (0.131)	0.567 (0.133)	0.986 (0.025)	0.062 (0.062)	0.615 (0.138)
**OR**	r	b	0.503 (0.237)	0.657 (0.100)	0.592 (0.189)	0.541 (0.337)	0.237 (0.237)	0.694 (0.132)
		u	0.259 (0.210)	0.511 (0.167)	0.481 (0.333)	0.391 (0.383)	0.079 (0.079)	0.610 (0.134)
	l	b	0.718 (0.126)	0.597 (0.125)	0.589 (0.149)	0.962 (0.043)	0.155 (0.155)	0.679 (0.116)
		u	0.646 (0.194)	0.675 (0.127)	0.638 (0.211)	0.713 (0.225)	0.338 (0.338)	0.739 (0.139)
**OX**	r	b	0.632 (0.185)	0.704 (0.106)	0.669 (0.171)	0.625 (0.220)	0.377 (0.377)	0.754 (0.127)
		u	0.599 (0.175)	0.694 (0.095)	0.641 (0.190)	0.594 (0.204)	0.357 (0.357)	0.748 (0.127)
	l	b	0.703 (0.153)	0.632 (0.129)	0.620 (0.153)	0.861 (0.202)	0.269 (0.269)	0.718 (0.183)
		u	0.658 (0.157)	0.594 (0.117)	0.577 (0.173)	0.813 (0.166)	0.202 (0.202)	0.661 (0.152)

Table 2: Mean and standard deviation (in parentheses) measures of F_1 score, classification accuracy, precision, recall, MCC and ROC-AUC at the local surface patch level obtained from the LOOCV procedure on the ***unbalanced training set*** using the corresponding best SVM model.

**Protein complex class**	**receptor ligand**	**bound un-bound**	**F_1 score**	**accuracy**	**precision**	**recall**	**MCC**	**ROC-AUC**
**A**	r	b	0.305 (0.100)	0.873 (0.027)	0.195 (0.083)	0.832 (0.178)	0.354 (0.354)	0.935 (0.048)
		u	0.300 (0.117)	0.872 (0.037)	0.198 (0.105)	0.802 (0.156)	0.343 (0.343)	0.919 (0.048)
	l	b	0.089 (0.063)	0.720 (0.173)	0.061 (0.046)	0.296 (0.226)	0.021 (0.021)	0.551 (0.044)
		u	0.095 (0.038)	0.198 (0.125)	0.051 (0.022)	0.867 (0.117)	0.011 (0.011)	0.525 (0.057)
**AB**	r	b	0.260 (0.119)	0.876 (0.077)	0.165 (0.079)	0.647 (0.259)	0.283 (0.283)	0.830 (0.178)
		u	0.239 (0.132)	0.870 (0.076)	0.158 (0.095)	0.570 (0.290)	0.249 (0.249)	0.801 (0.203)
	l	b	0.136 (0.078)	0.605 (0.067)	0.082 (0.052)	0.522 (0.152)	0.067 (0.067)	0.597 (0.061)
		u	0.124 (0.057)	0.229 (0.059)	0.068 (0.034)	0.944 (0.039)	0.078 (0.078)	0.641 (0.085)
**EI**	r	b	0.200 (0.083)	0.636 (0.107)	0.121 (0.068)	0.756 (0.153)	0.190 (0.190)	0.764 (0.104)
		u	0.162 (0.081)	0.658 (0.096)	0.096 (0.050)	0.623 (0.270)	0.137 (0.137)	0.698 (0.167)
	l	b	0.315 (0.139)	0.485 (0.121)	0.200 (0.109)	0.896 (0.115)	0.218 (0.218)	0.782 (0.143)
		u	0.279 (0.142)	0.328 (0.091)	0.184 (0.155)	0.946 (0.096)	0.138 (0.138)	0.711 (0.081)
**ER**	r	b	0.131 (0.055)	0.720 (0.059)	0.076 (0.035)	0.580 (0.136)	0.125 (0.125)	0.708 (0.084)
		u	0.117 (0.060)	0.740 (0.064)	0.068 (0.037)	0.529 (0.200)	0.110 (0.110)	0.692 (0.127)
	l	b	0.227 (0.120)	0.473 (0.089)	0.140 (0.101)	0.861 (0.094)	0.173 (0.173)	0.738 (0.072)
		u	0.153 (0.085)	0.093 (0.057)	0.085 (0.054)	1.000 (0.000)	0.024 (0.024)	0.700 (0.124)
**ES**	r	b	0.083 (0.053)	0.696 (0.296)	0.077 (0.098)	0.407 (0.324)	0.063 (0.063)	0.661 (0.085)
		u	0.065 (0.081)	0.748 (0.343)	0.044 (0.052)	0.402 (0.381)	0.069 (0.069)	0.687 (0.100)
	l	b	0.223 (0.136)	0.669 (0.075)	0.145 (0.109)	0.643 (0.119)	0.180 (0.180)	0.698 (0.097)
		u	0.172 (0.083)	0.621 (0.047)	0.099 (0.051)	0.731 (0.156)	0.162 (0.162)	0.716 (0.100)
**OG**	r	b	0.106 (0.047)	0.714 (0.079)	0.063 (0.033)	0.492 (0.225)	0.087 (0.087)	0.672 (0.130)
		u	0.093 (0.047)	0.824 (0.053)	0.058 (0.033)	0.315 (0.122)	0.069 (0.069)	0.645 (0.090)
	l	b	0.175 (0.070)	0.348 (0.088)	0.099 (0.044)	0.886 (0.107)	0.110 (0.110)	0.715 (0.123)
		u	0.127 (0.055)	0.085 (0.039)	0.069 (0.031)	0.985 (0.024)	0.012 (0.012)	0.594 (0.145)
**OR**	r	b	0.104 (0.048)	0.619 (0.298)	0.062 (0.030)	0.529 (0.329)	0.067 (0.067)	0.635 (0.140)
		u	0.049 (0.050)	0.608 (0.359)	0.048 (0.068)	0.375 (0.373)	0.005 (0.005)	0.529 (0.131)
	l	b	0.139 (0.067)	0.168 (0.066)	0.076 (0.040)	0.967 (0.038)	0.060 (0.060)	0.658 (0.120)
		u	0.165 (0.125)	0.560 (0.120)	0.100 (0.084)	0.695 (0.244)	0.127 (0.127)	0.680 (0.153)
**OX**	r	b	0.160 (0.102)	0.689 (0.054)	0.096 (0.068)	0.611 (0.216)	0.140 (0.140)	0.700 (0.132)
		u	0.135 (0.094)	0.674 (0.061)	0.081 (0.063)	0.575 (0.215)	0.110 (0.110)	0.670 (0.138)
	l	b	0.148 (0.072)	0.359 (0.110)	0.083 (0.044)	0.857 (0.202)	0.089 (0.089)	0.698 (0.184)
		u	0.126 (0.066)	0.340 (0.086)	0.070 (0.039)	0.801 (0.169)	0.057 (0.057)	0.611 (0.149)

4 Post-processing

4.1 Best SVM threshold values

We noticed that for some protein classes the prediction performance in terms of ROC-AUC was high while the other prediction metrics were low. This is due to the fact that the default threshold ($t = 0$) used by the SVM classifier does not yield optimal binary classification results, since the employed training set is balanced and does not reflect the natural distribution of interface and non-interface patches. We selected the best threshold value that maximises the average F_1 score on the training set proteins for each protein class: we used the unbalanced version of the test set for this task. The results are reported in Figs. 1 to 8.

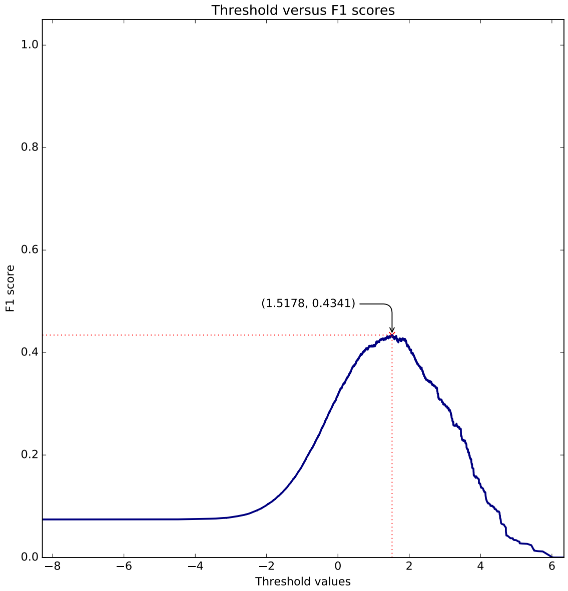

(a) A:r:u

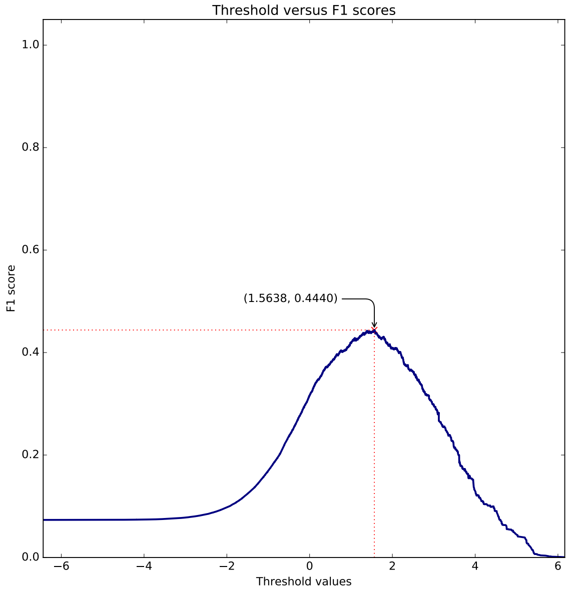

(b) A:r:b

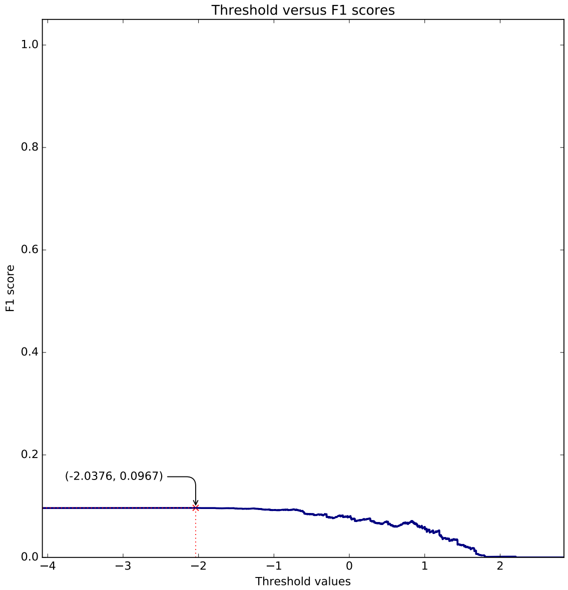

(c) A:l:u

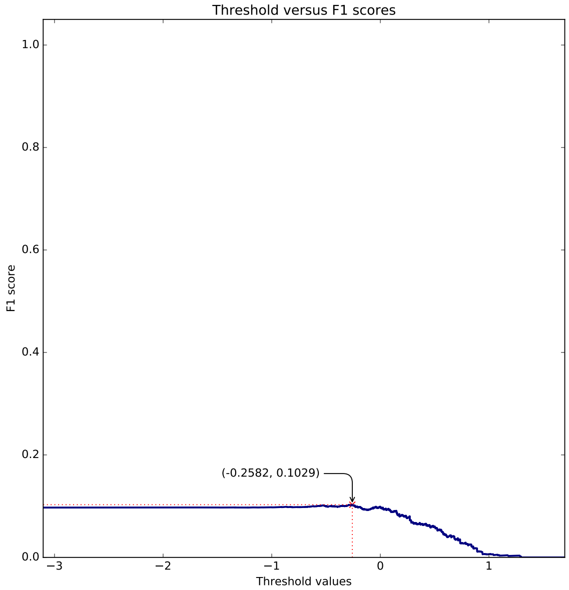

(d) A:l:b

Figure 1: SVM threshold vs F_1 score plot on the unbalanced version of the training set at the local surface patch level for protein class A.

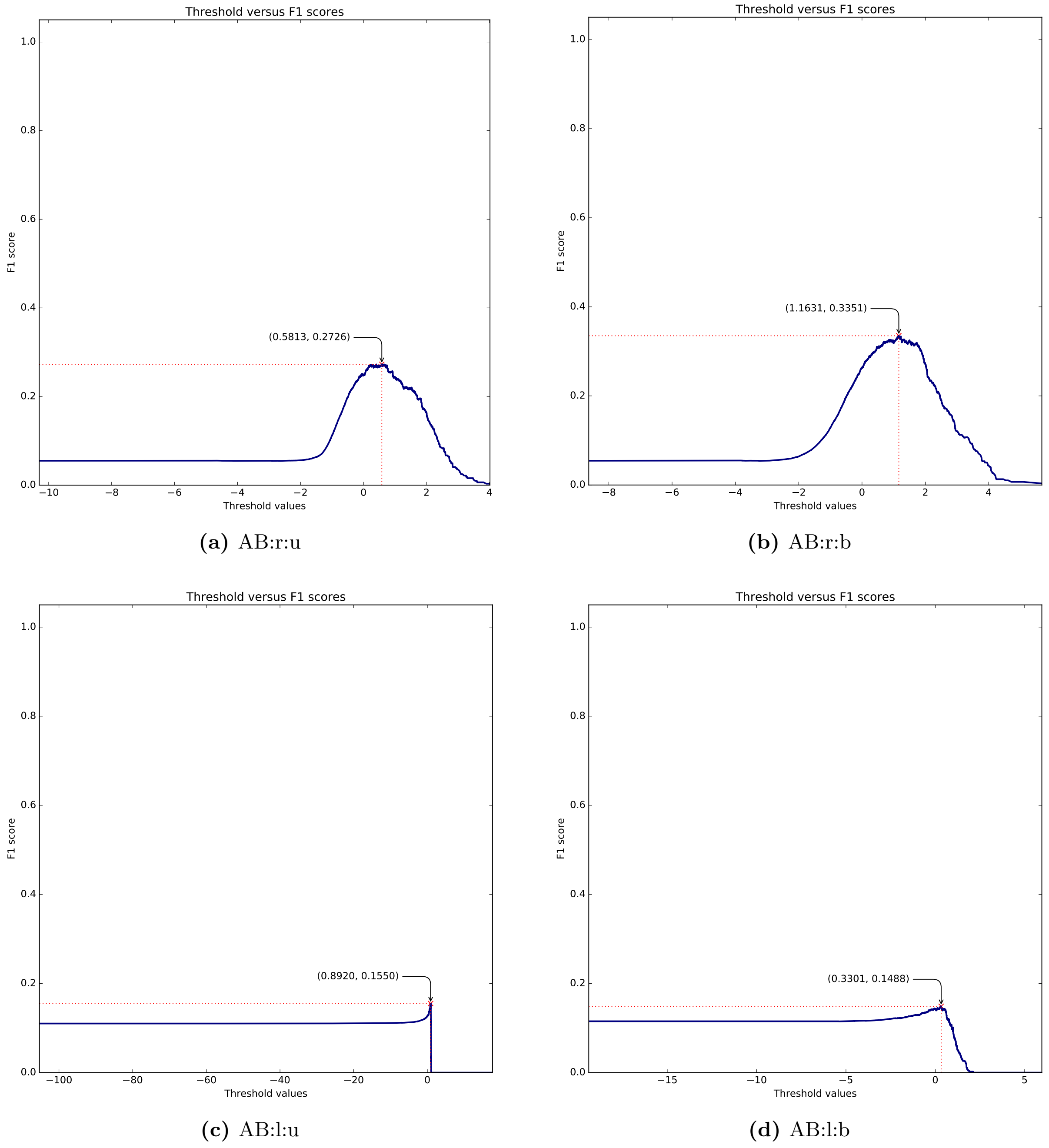

Figure 2: SVM threshold vs F_1 score plot on the unbalanced version of the training set at the local surface patch level for protein class AB.

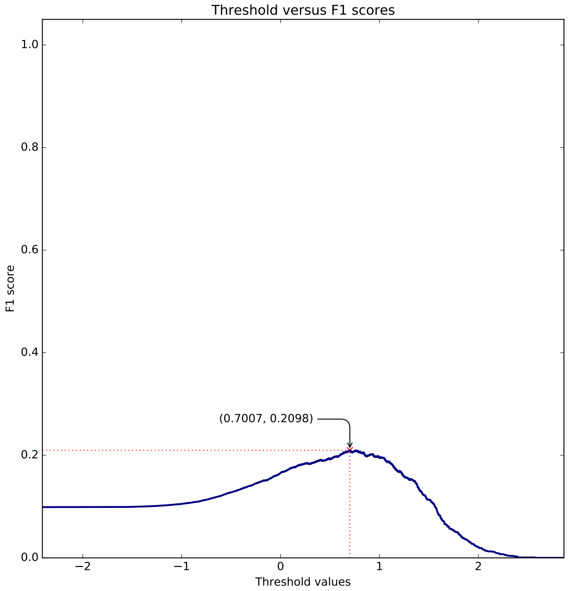

(a) EI:r:u

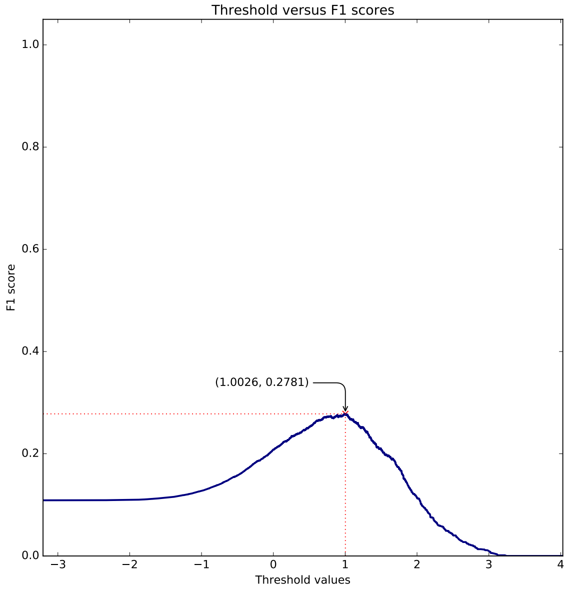

(b) EI:r:b

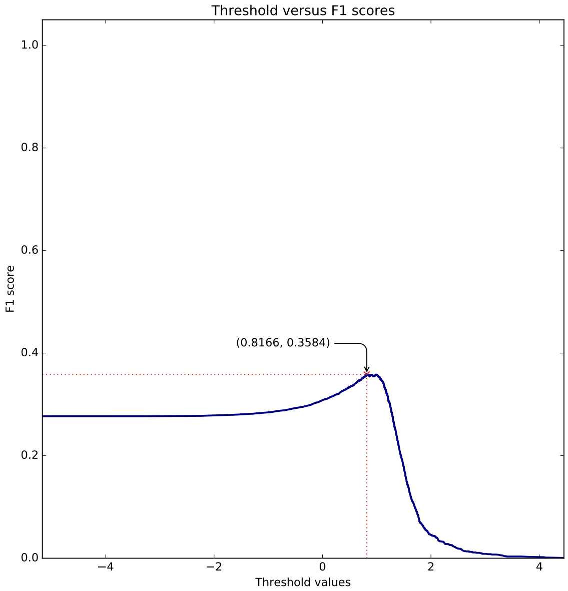

(c) EI:l:u

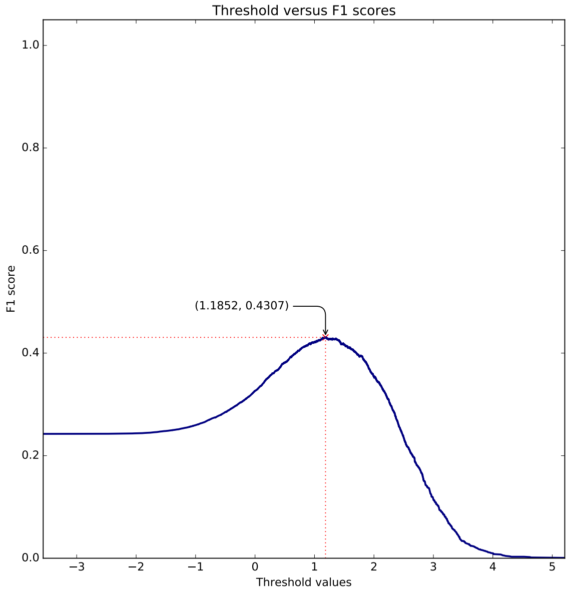

(d) EI:l:b

Figure 3: SVM threshold vs F_1 score plot on the unbalanced version of the training set at the local surface patch level for protein class EI.

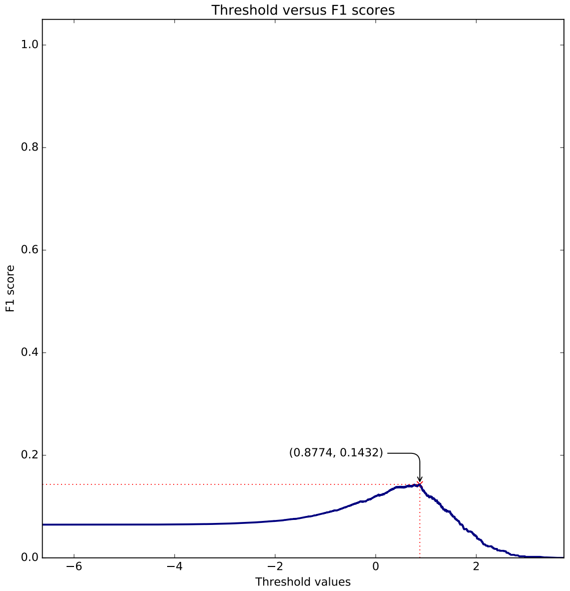

(a) ER:r:u

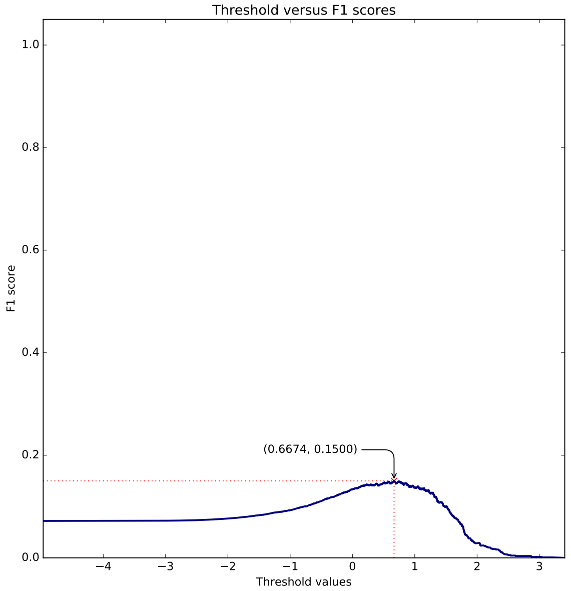

(b) ER:r:b

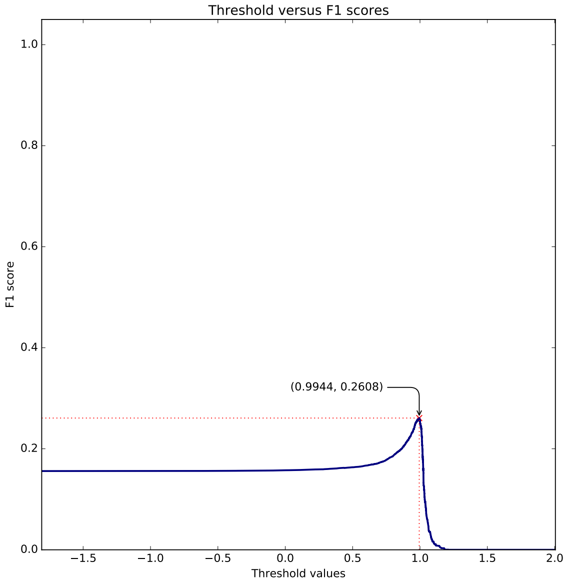

(c) ER:l:u

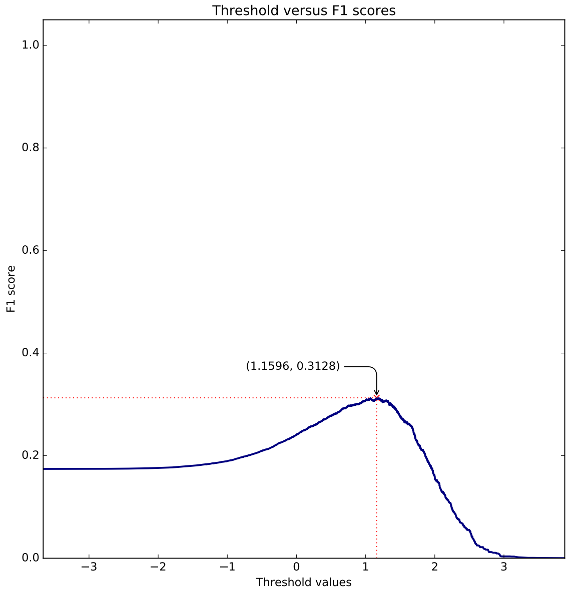

(d) ER:l:b

Figure 4: SVM threshold vs F_1 score plot on the unbalanced version of the training set at the local surface patch level for protein class ER.

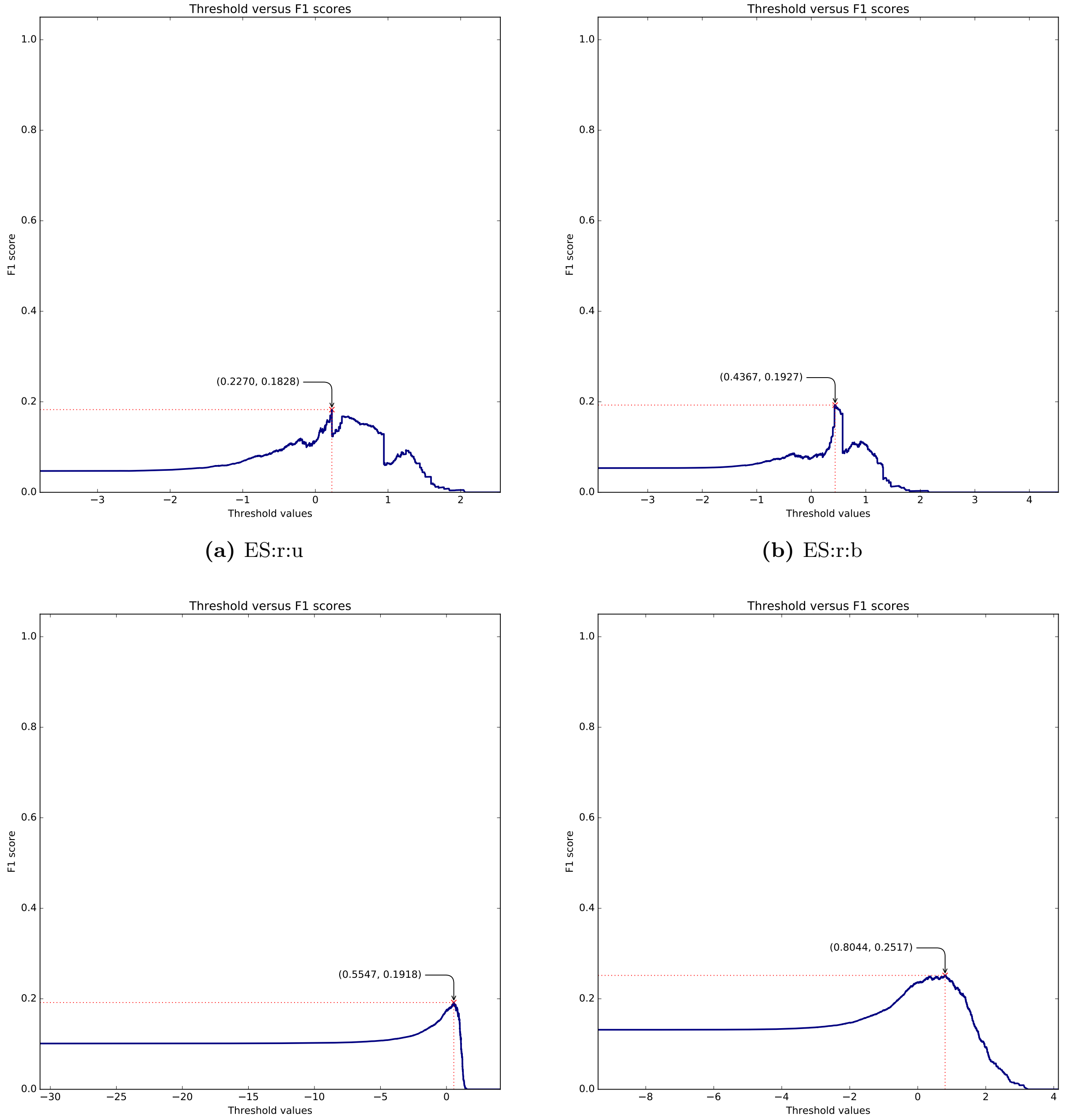

(a) ES:r:u

(b) ES:r:b

(c) ES:l:u

(d) ES:l:b

Figure 5: SVM threshold vs F_1 score plot on the unbalanced version of the training set at the local surface patch level for protein class ES.

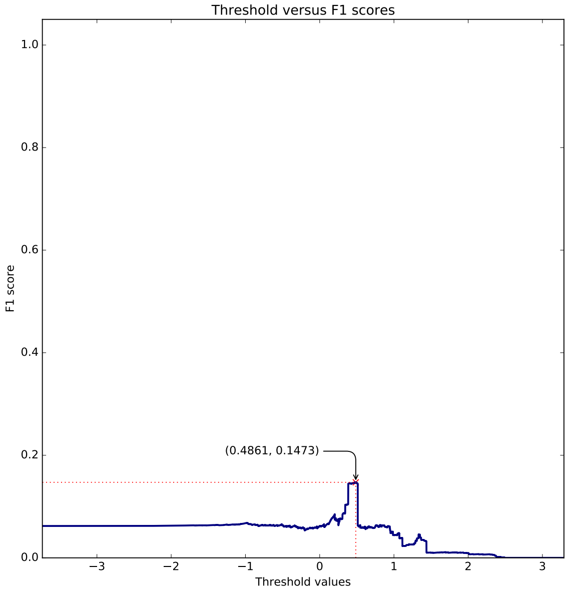

(a) OR:r:u

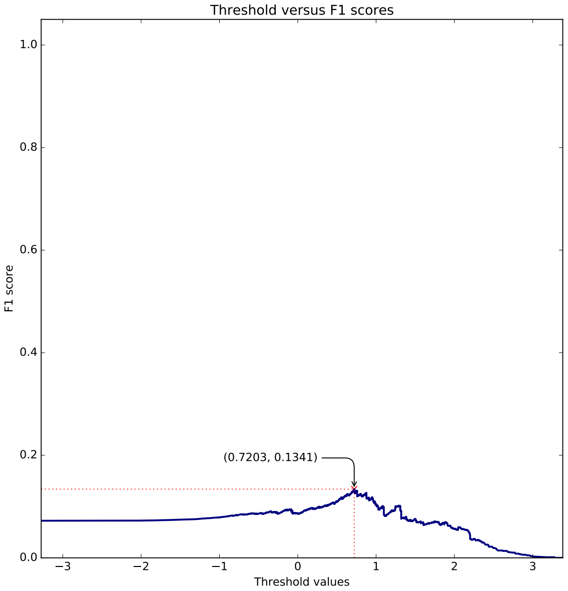

(b) OR:r:b

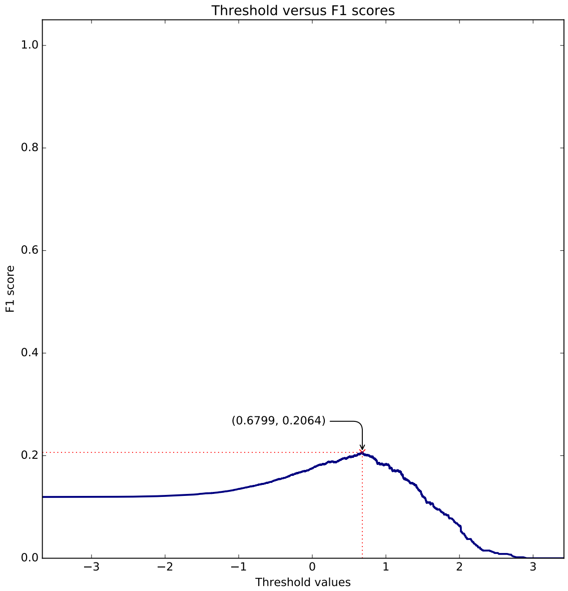

(c) OR:l:u

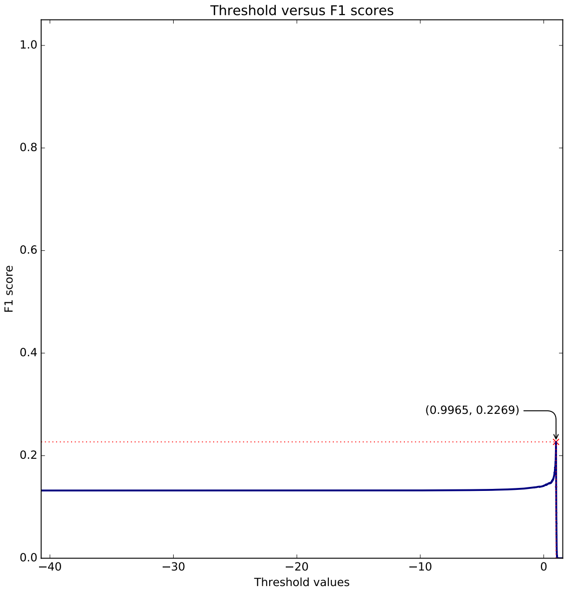

(d) OR:l:b

Figure 6: SVM threshold vs F_1 score plot on the unbalanced version of the training set at the local surface patch level for protein class OR.

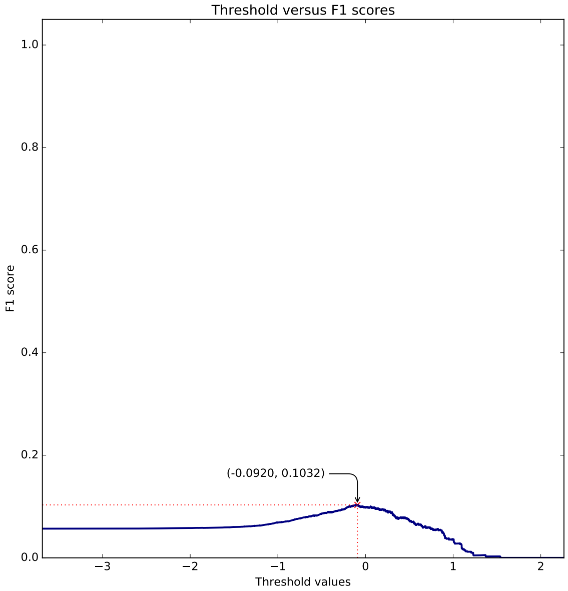

(a) OG:r:u

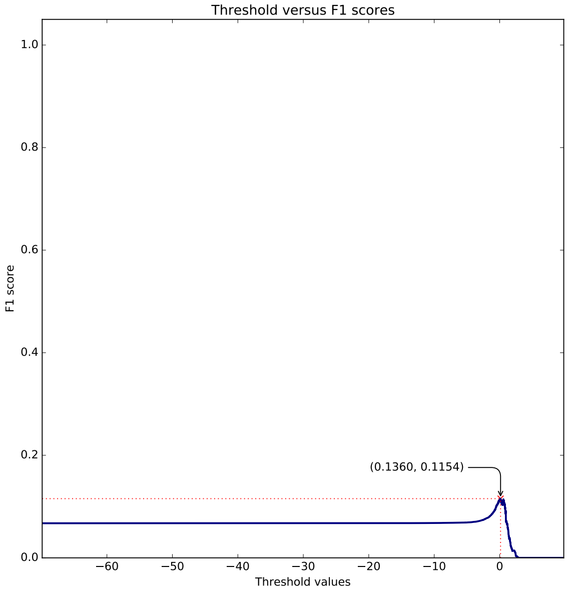

(b) OG:r:b

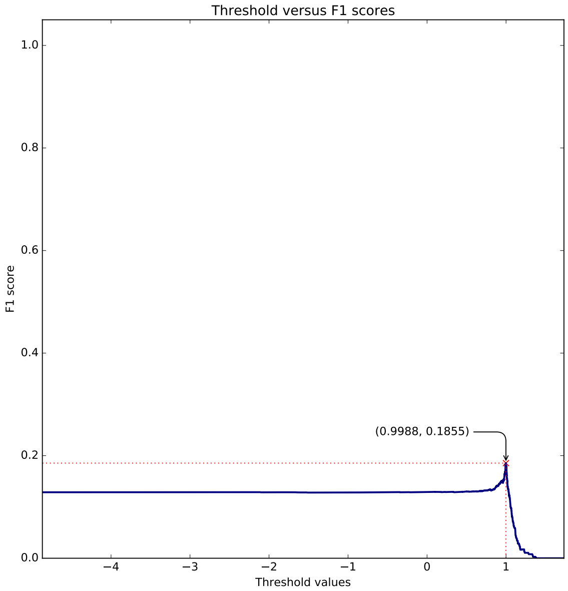

(c) OG:l:u

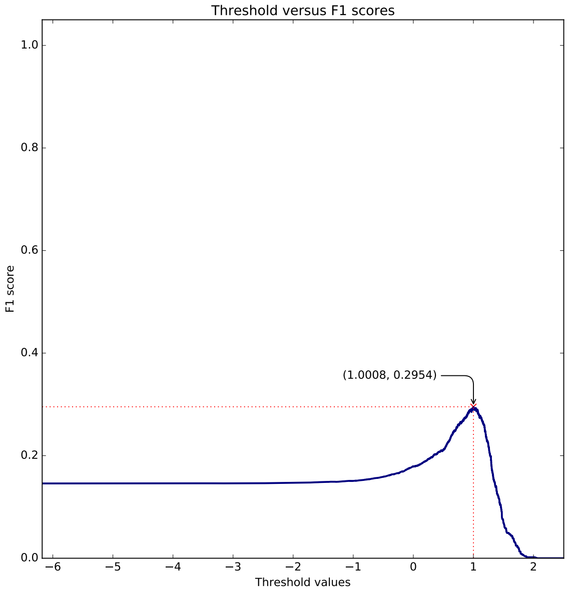

(d) OG:l:b

Figure 7: SVM threshold vs F_1 score plot on the unbalanced version of the training set at the local surface patch level for protein class OG.

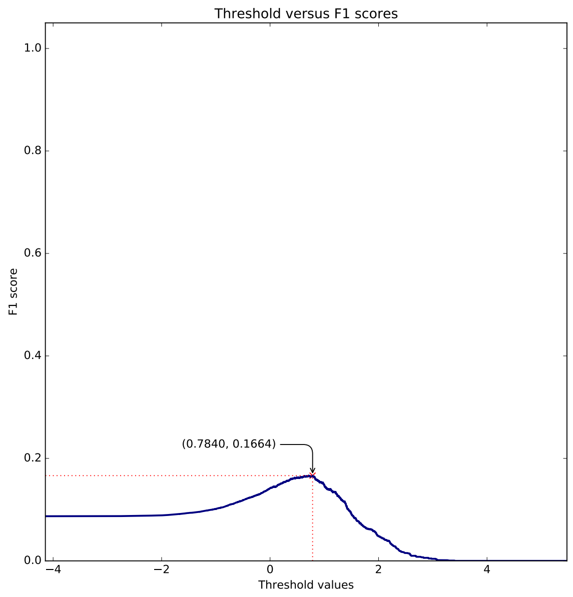

(a) OX:r:u

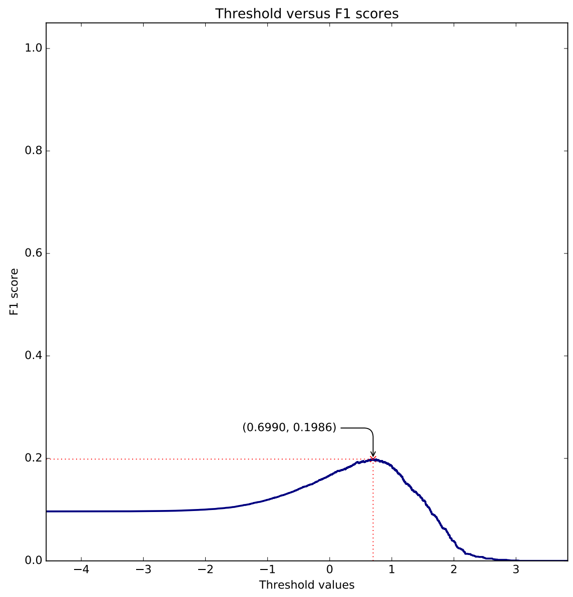

(b) OX:r:b

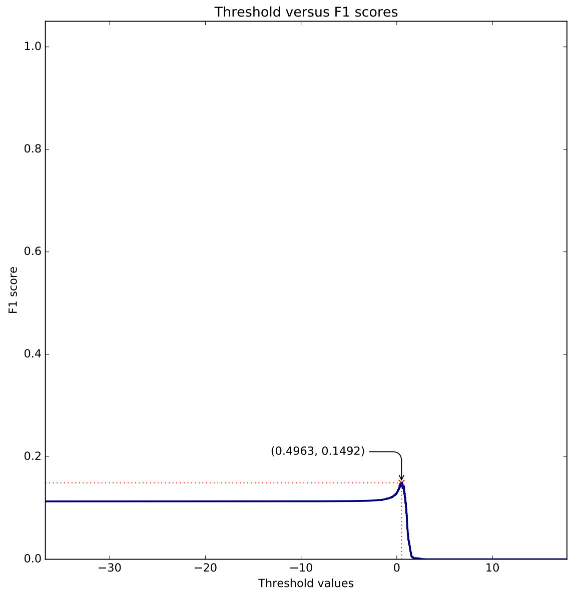

(c) OX:l:u

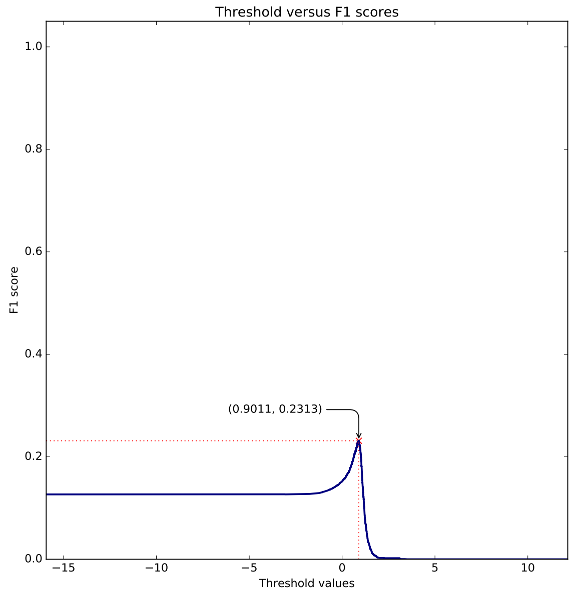

(d) OX:l:b

Figure 8: SVM threshold vs F_1 score plot on the unbalanced version of the training set at the local surface patch level for protein class OX.

4.2 Best contamination values

The Isolation Forest (IF) algorithm for outlier detection [5] was used to reduce the number of spatially-isolated false positive local surface patches. Interface regions are composed of contiguous surface patches, thus isolated patches marked as positive by the SVM classifier can be safely discarded. For each query protein, an IF classifier is trained on the coordinates of the LSPs identified as interface patches by the SVM classifier, using their distances from the separating hyperplane as weights. Then, the IF classifier is used on the whole set of surface patches of the query protein to identify the ones belonging to the PPI interface. A contamination parameter must be provided to the IF algorithm: we identified the optimal parameter values for each protein class by testing all contamination values from 0.00 to 0.5 with a constant increment of 0.01, and selected the ones that yielded the best average F_1 score on the training set of the corresponding protein class. Because the IF for outlier detection is a random algorithm, the F_1 score was averaged over 100 runs for each contamination value. When the best average F_1 score was

obtained for a contamination value equal to zero we skipped the IF step. The results are reported in Figs. 9 to 16.

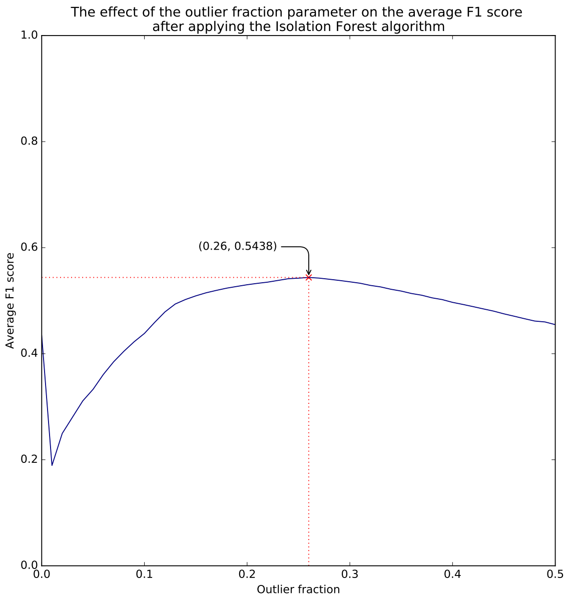

(a) A:r:u

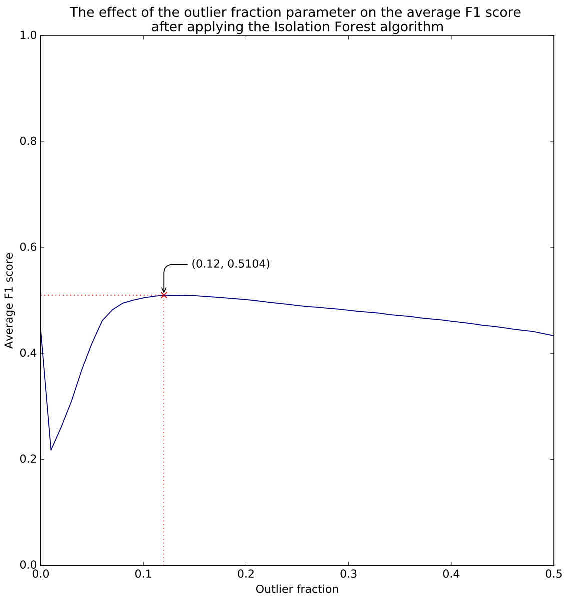

(b) A:r:b

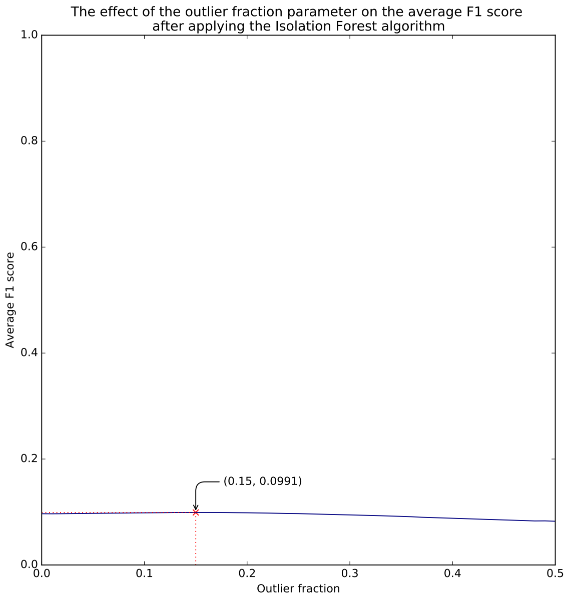

(c) A:l:u

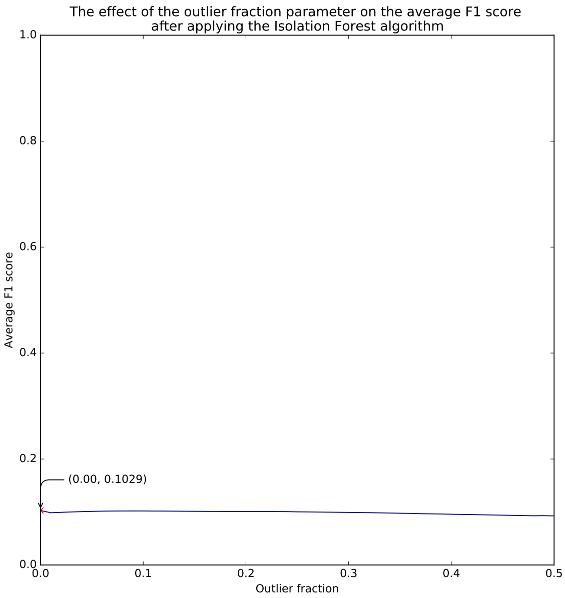

(d) A:l:b

Figure 9: Contamination (outlier fraction) vs F_1 score plot on the unbalanced version of the training set at the local surface patch level for protein class A.

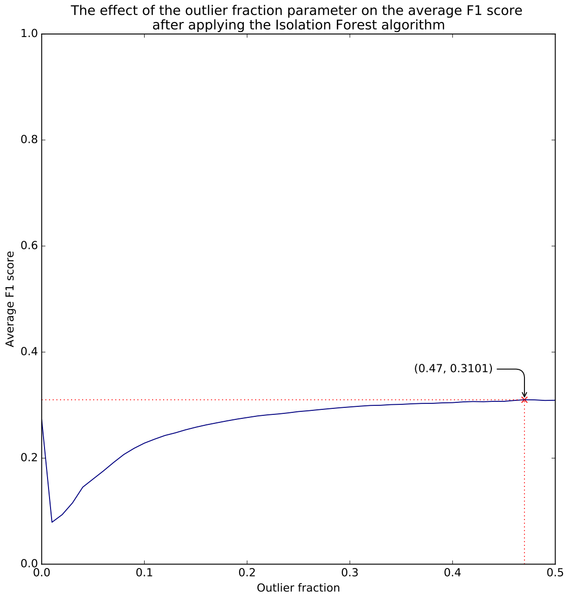

(a) AB:r:u

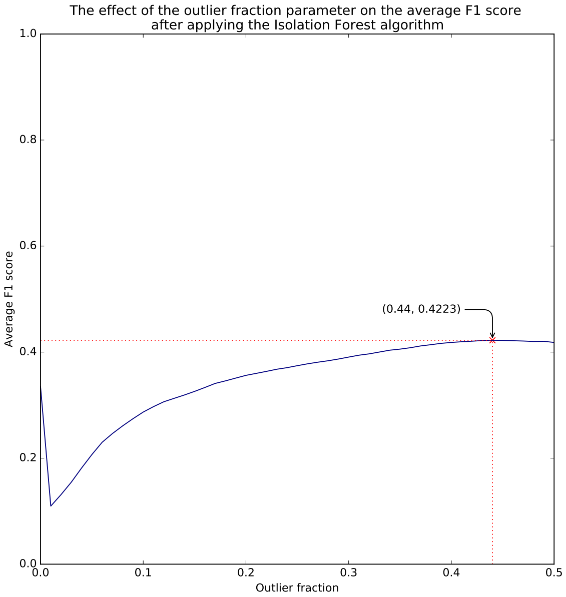

(b) AB:r:b

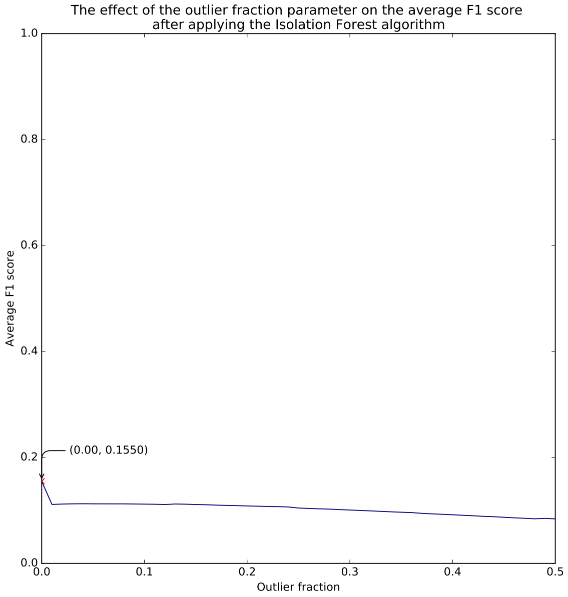

(c) AB:l:u

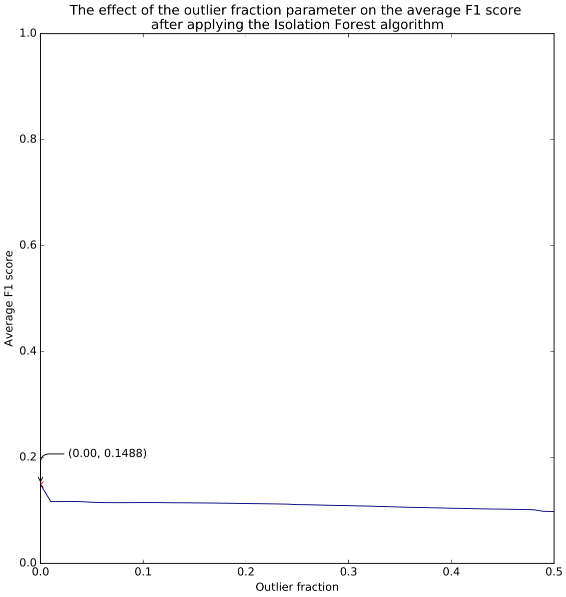

(d) AB:l:b

Figure 10: Contamination (outlier fraction) vs F_1 score plot on the unbalanced version of the training set at the local surface patch level for protein class AB.

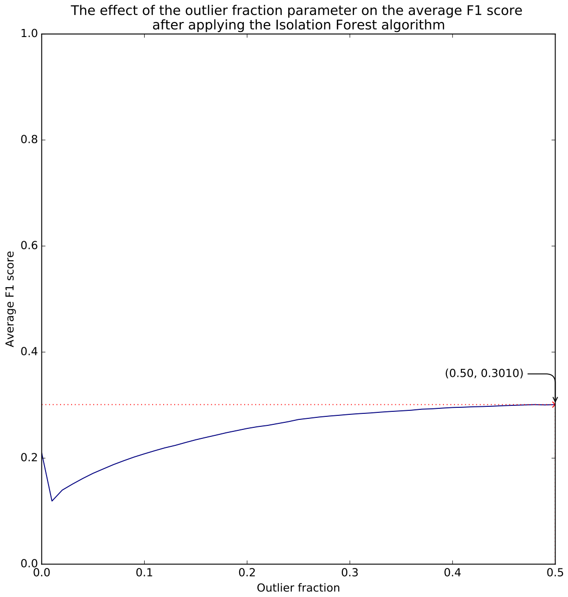

(a) EI:r:u

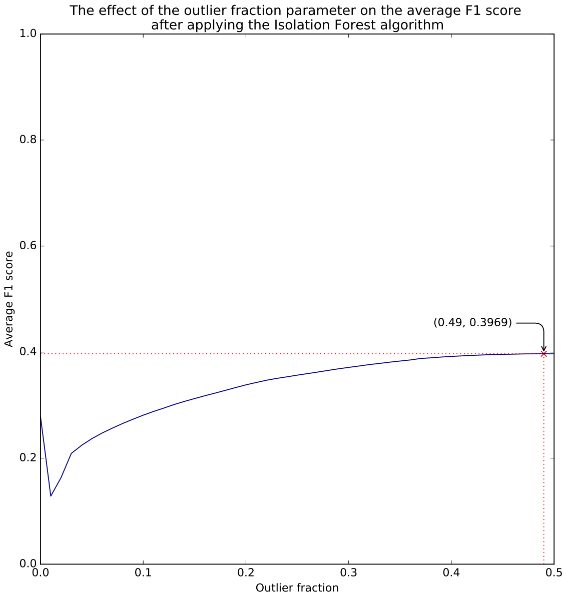

(b) EI:r:b

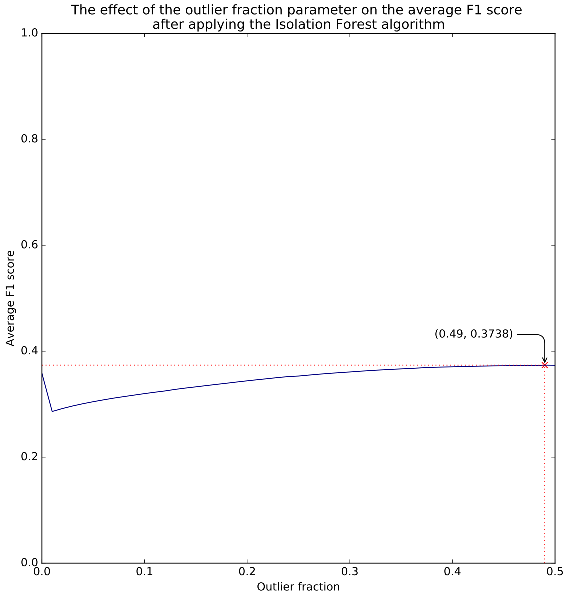

(c) EI:l:u

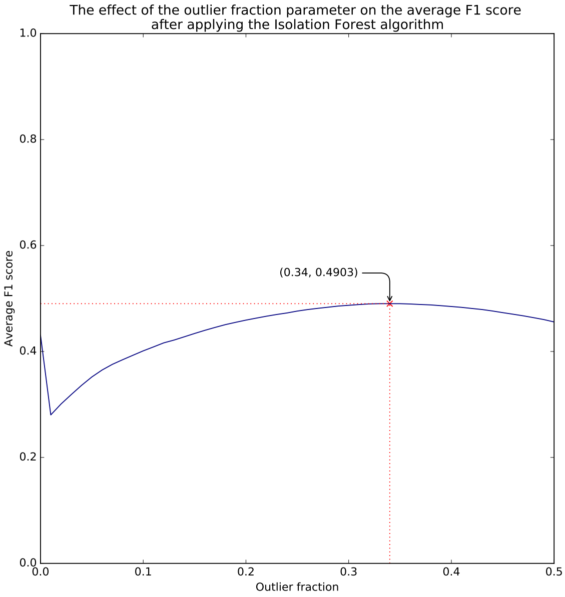

(d) EI:l:b

Figure 11: Contamination (outlier fraction) vs F_1 score plot on the unbalanced version of the training set at the local surface patch level for protein class EI.

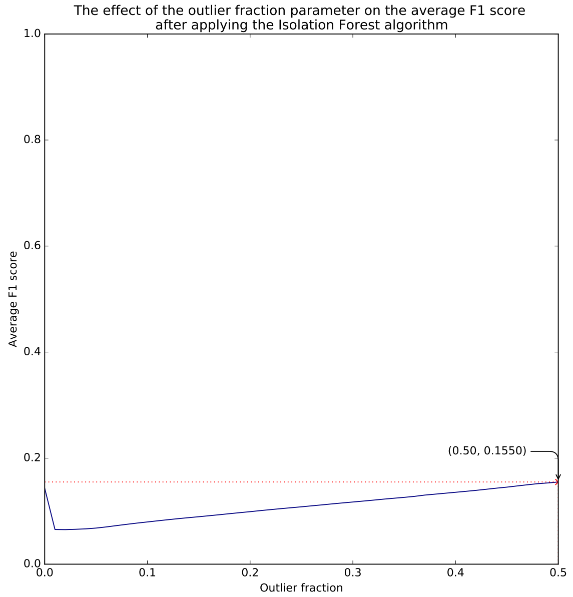

(a) ER:r:u

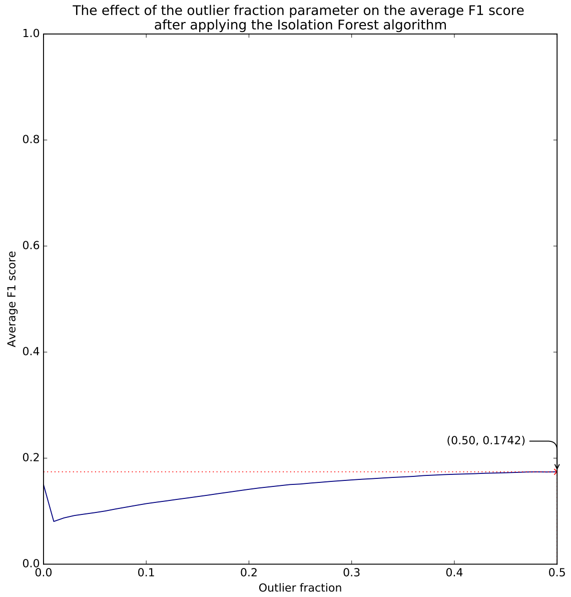

(b) ER:r:b

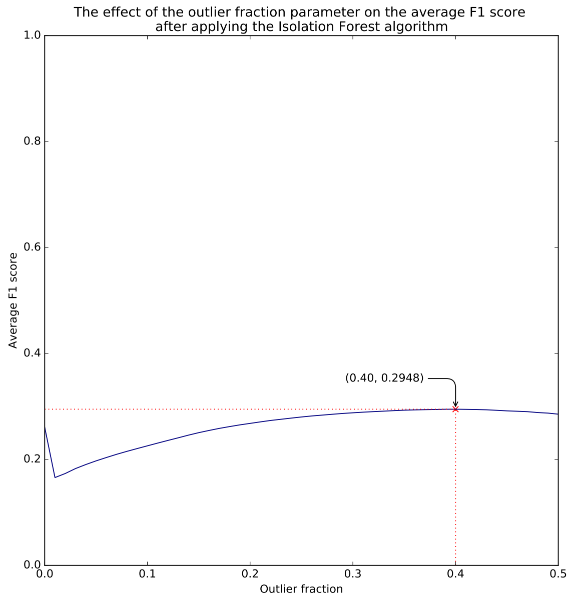

(c) ER:l:u

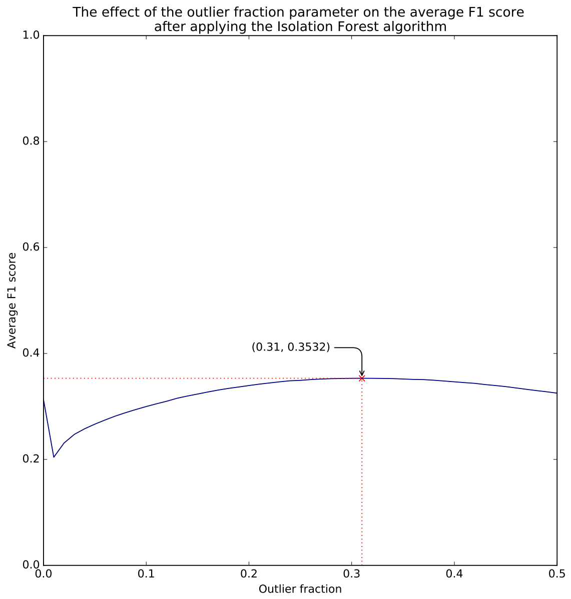

(d) ER:l:b

Figure 12: Contamination (outlier fraction) vs F_1 score plot on the unbalanced version of the training set at the local surface patch level for protein class ER.

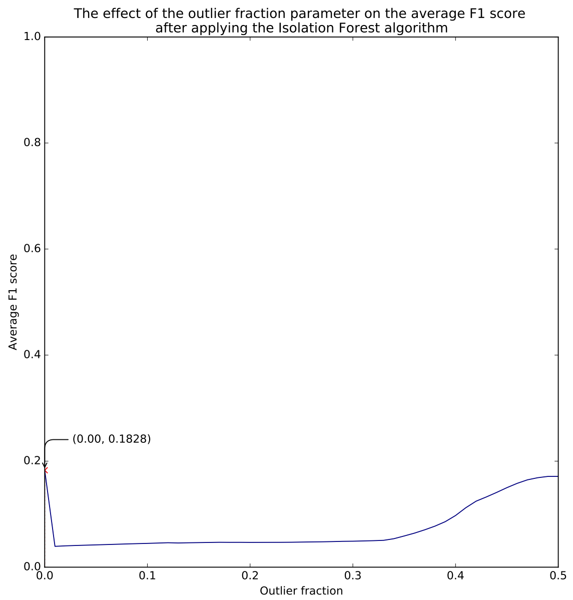

(a) ES:r:u

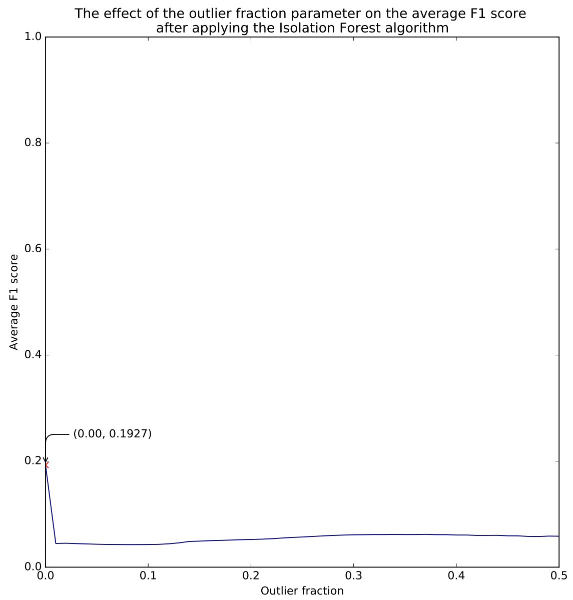

(b) ES:r:b

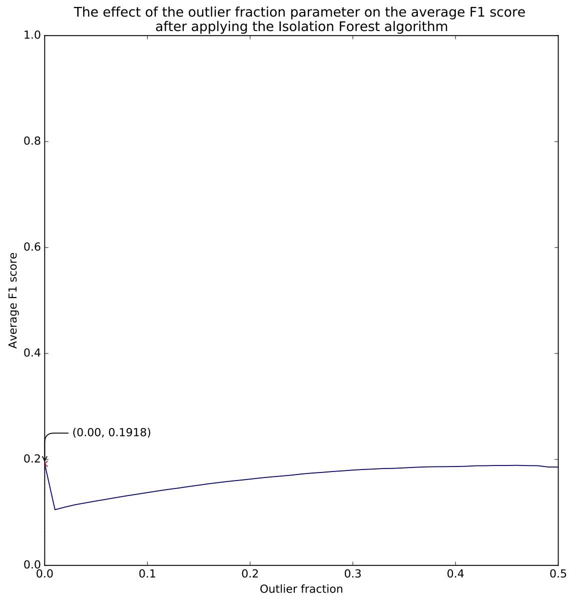

(c) ES:l:u

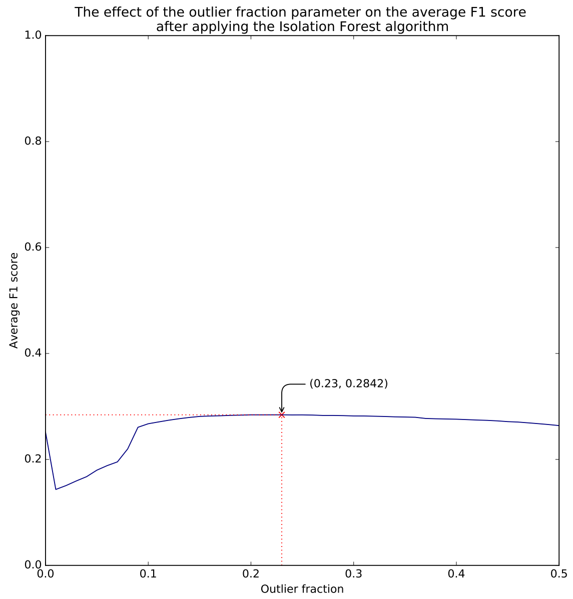

(d) ES:l:b

Figure 13: Contamination (outlier fraction) vs F_1 score plot on the unbalanced version of the training set at the local surface patch level for protein class ES.

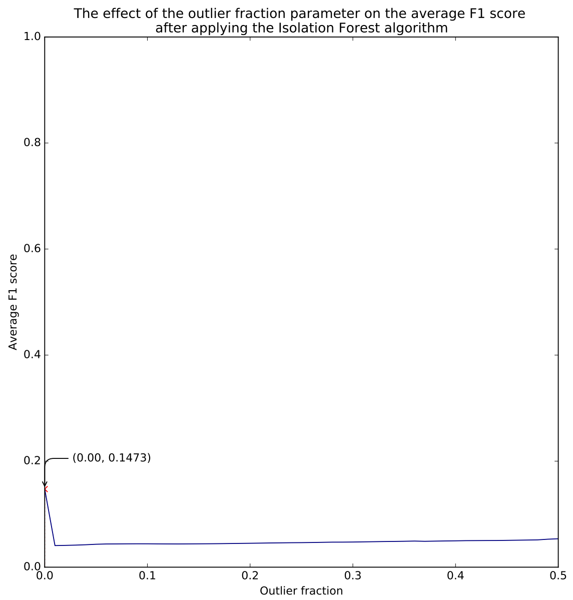

(a) OR:r:u

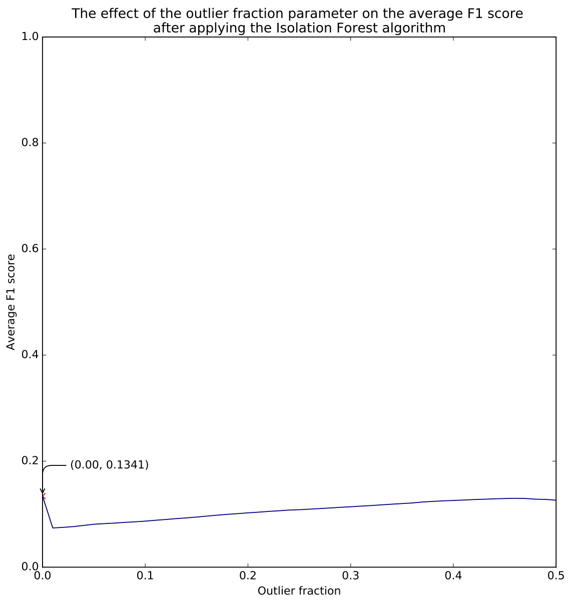

(b) OR:r:b

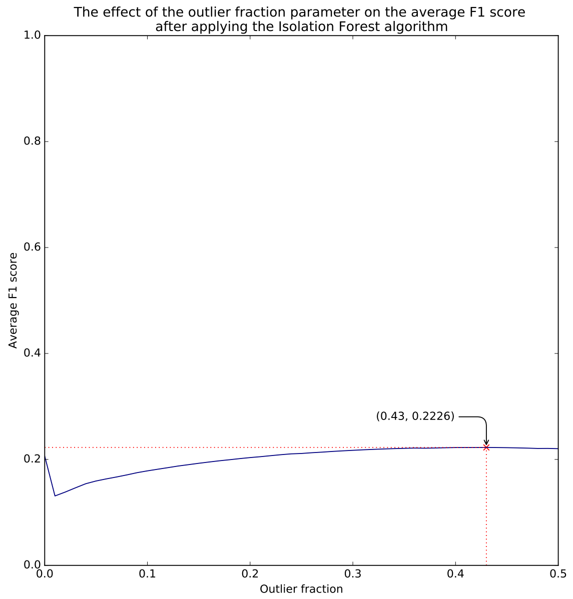

(c) OR:l:u

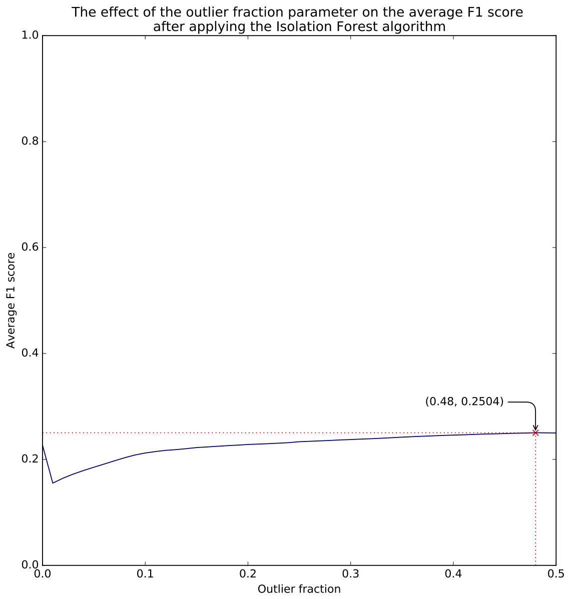

(d) OR:l:b

Figure 14: Contamination (outlier fraction) vs F_1 score plot on the unbalanced version of the training set at the local surface patch level for protein class OR.

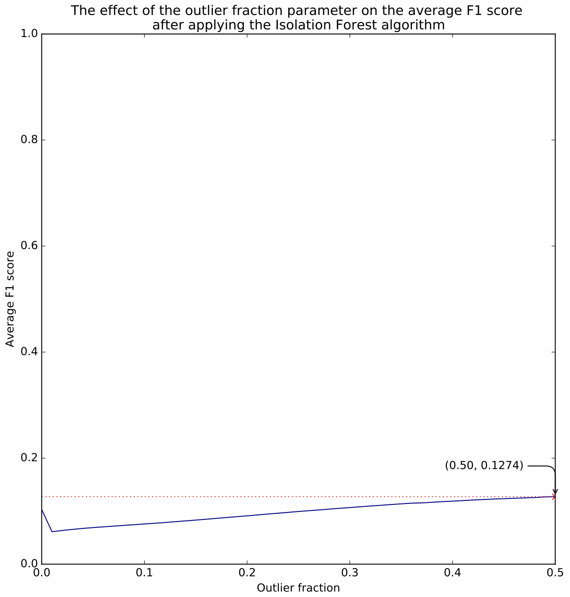

(a) OG:r:u

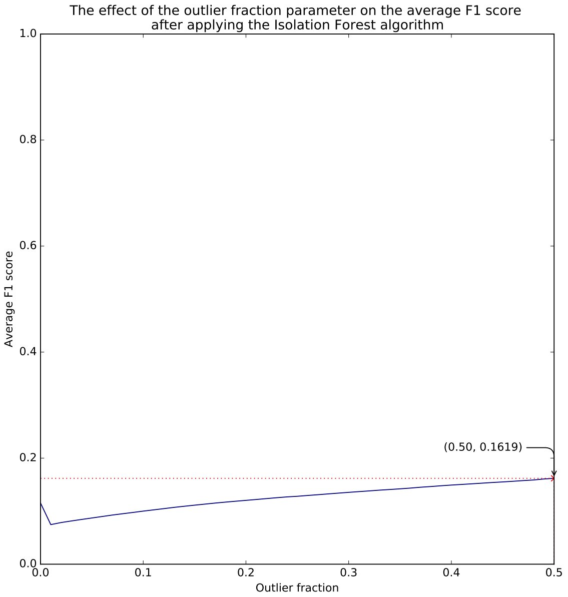

(b) OG:r:b

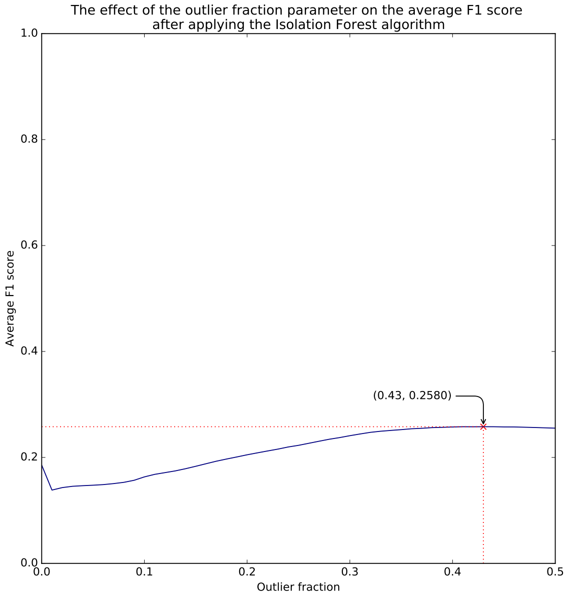

(c) OG:l:u

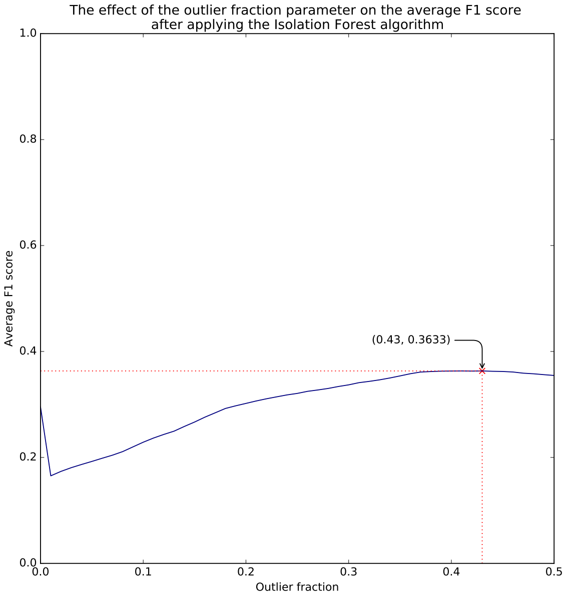

(d) OG:l:b

Figure 15: Contamination (outlier fraction) vs F_1 score plot on the unbalanced version of the training set at the local surface patch level for protein class OG.

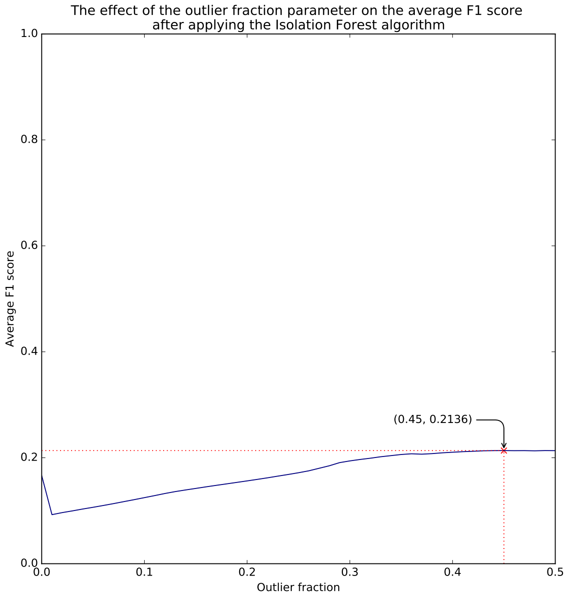

(a) OX:r:u

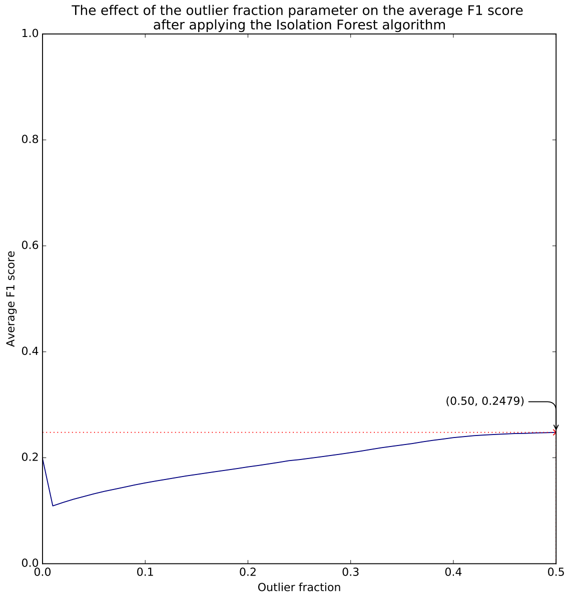

(b) OX:r:b

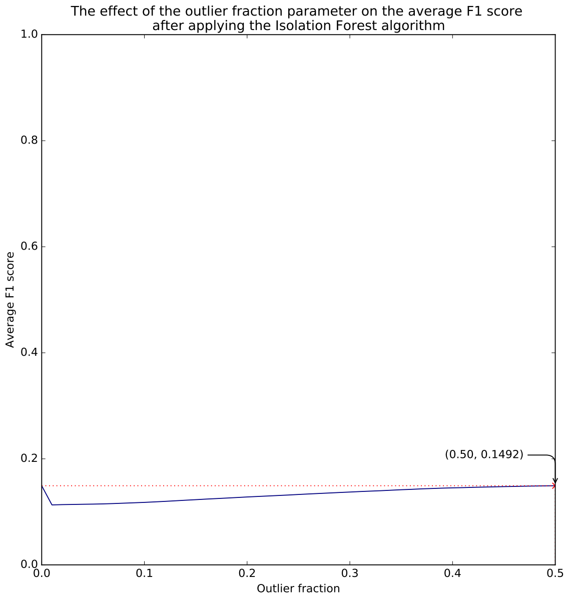

(c) OX:l:u

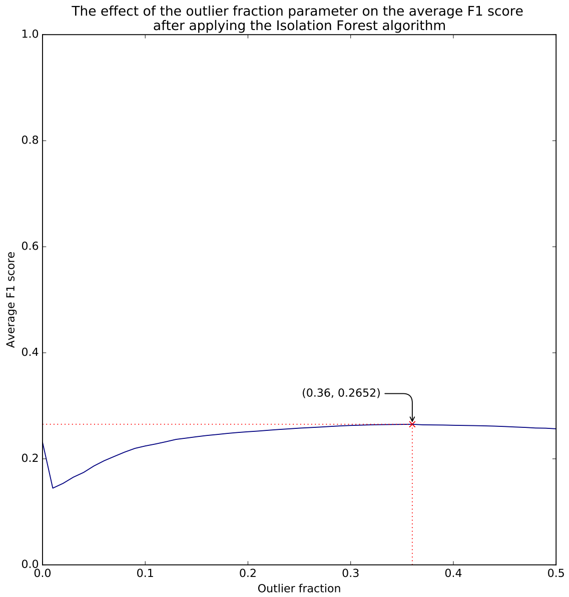

(d) OX:l:b

Figure 16: Contamination (outlier fraction) vs F_1 score plot on the unbalanced version of the training set at the local surface patch level for protein class OX.

References

[1] N. Canterakis, "3D Zernike Moments and Zernike Affine Invariants for 3D Image Analysis and Recognition," in *In 11th Scandinavian Conf. on Image Analysis*, B. Ersbøll and P. Johansen, Eds. Kangerlussuaq, Greenland: Dansk Selskab for Automatisk Genkendelse af Mønstre, 1999, pp. 85–93.

[2] M. Novotni and R. Klein, "Shape retrieval using 3D Zernike descriptors," *Computer-Aided Design*, vol. 36, no. 11, pp. 1047–1062, 2004.

[3] G. James, D. Witten, T. Hastie, and R. Tibshirani, *An introduction to statistical learning.* New York, NY, USA: Springer, 2013, vol. 6.

[4] N. Meinshausen and P. Bühlmann, "Stability selection," *Journal of the Royal Statistical Society: Series B (Statistical Methodology)*, vol. 72, no. 4, pp. 417–473, 2010.

[5] F. T. Liu *et al.*, "Isolation-based anomaly detection," *ACM Trans. Knowl. Discov. Data*, vol. 6, no. 1, p. 3, 2012.
